# Loss of the ribosomal RNA methyltransferase NSUN5 impairs global protein synthesis and normal growth

**DOI:** 10.1093/nar/gkz1043

**Published:** 2019-11-13

**Authors:** Clemens Heissenberger, Lisa Liendl, Fabian Nagelreiter, Yulia Gonskikh, Guohuan Yang, Elena M Stelzer, Teresa L Krammer, Lucia Micutkova, Stefan Vogt, David P Kreil, Gerhard Sekot, Emilio Siena, Ina Poser, Eva Harreither, Angela Linder, Viktoria Ehret, Thomas H Helbich, Regina Grillari-Voglauer, Pidder Jansen-Dürr, Martin Koš, Norbert Polacek, Johannes Grillari, Markus Schosserer

**Affiliations:** 1 Department of Biotechnology, Institute of Molecular Biotechnology, University of Natural Resources and Life Sciences, Vienna, 1190 Vienna, Austria; 2 Department of Chemistry and Biochemistry, University of Bern, 3012 Bern, Switzerland; 3 Biochemistry Center, University of Heidelberg, 69120 Heidelberg, Germany; 4 Institute for Biomedical Aging Research, University of Innsbruck, 6020 Innsbruck, Austria; 5 Max Planck Institute for Molecular Cell Biology and Genetics, 01307 Dresden, Germany; 6 Department of Biomedical Imaging and Image-guided Therapy, Division of Molecular and Gender Imaging, Preclinical Imaging Laboratory, Medical University of Vienna, 1090 Vienna, Austria; 7 Christian Doppler Laboratory on Biotechnology of Skin Aging, 1190 Vienna, Austria; 8 Ludwig Boltzmann Institute for Experimental and Clinical Traumatology, 1200 Vienna, Austria

## Abstract

Modifications of ribosomal RNA expand the nucleotide repertoire and thereby contribute to ribosome heterogeneity and translational regulation of gene expression. One particular m^5^C modification of 25S ribosomal RNA, which is introduced by Rcm1p, was previously shown to modulate stress responses and lifespan in yeast and other small organisms. Here, we report that NSUN5 is the functional orthologue of Rcm1p, introducing m^5^C3782 into human and m^5^C3438 into mouse 28S ribosomal RNA. Haploinsufficiency of the NSUN5 gene in fibroblasts from William Beuren syndrome patients causes partial loss of this modification. The N-terminal domain of NSUN5 is required for targeting to nucleoli, while two evolutionary highly conserved cysteines mediate catalysis. Phenotypic consequences of NSUN5 deficiency in mammalian cells include decreased proliferation and size, which can be attributed to a reduction in total protein synthesis by altered ribosomes. Strikingly, Nsun5 knockout in mice causes decreased body weight and lean mass without alterations in food intake, as well as a trend towards reduced protein synthesis in several tissues. Together, our findings emphasize the importance of single RNA modifications for ribosome function and normal cellular and organismal physiology.

## INTRODUCTION

Control of cell size and proliferation are integral, but clearly separated processes with many underlying mechanisms remaining unexplained. Our current understanding is that both intrinsic developmental programs and extracellular signals control cell number and cell size (1), ultimately contributing to growth of whole organisms (2). Extracellular signals, such as genome size, metabolism, or nutrient availability and -uptake, impinge on important cellular signalling hubs such as the mTOR pathway (3) or the transcription factor Myc (4) that simultaneously control various anabolic processes. Protein synthesis is one of those and requires tight regulation by mTOR and Myc as energy demands are exceptionally high (5).

The conversion of mRNA blueprints to functional polypeptide chains is carried out by ribosomes. These large molecular machines are composed of proteins and RNA and were formerly seen as a static and homogenous population, performing protein synthesis in a constitutive manner. In recent years, however, it became evident that mammalian ribosomes rather represent dynamic structures that can respond to certain stimuli by adapting their structure and function (6,7). This heterogeneity can be generated by changes in stoichiometry and modification patterns of any of the ∼80 core ribosomal proteins (RPs), or their association with other factors. Importantly, 2–3% of the 7000 nucleotides distributed over the four ribosomal RNAs (rRNA) are decorated with post-transcriptional modifications (8), which are introduced either by specific small nucleolar RNA (snoRNA)-driven or stand-alone snoRNA-independent enzymes (8,9). RNA modifications expand the naturally limited properties of the four nucleotides and thereby contribute to ribosome heterogeneity by adapting rRNA stability, structure and function. Apart from more abundant 2′-*O*-ribose methyls and pseudouridines (Ψ), only five types of base modifications, namely acetylated cytosine (ac^4^C) and *N*,*N*-dimethyladenosine (m^6^_2_A) on the small subunit (SSU), as well as *N*^1^-methyladenosine (m^1^A), *N*^3^-methyluridine (m^3^U) and *C*^5^-methylcytosine (m^5^C) on the large subunit (LSU) are each present twice per ribosome in yeast, indicating a particularly beneficial role in terms of chemical stability and structure (10). Similarly, according to current estimations human 18S rRNA carries two ac^4^C, two m^6^_2_A, one N-methylguanosine (m^7^G) and one *N*-methyl-*N*-aminocarboxypropylpseudouridine (m^1^acp^3^ψ), while 28S rRNA is decorated with one m^1^A, two m^5^C and two m^3^U. Intriguingly, the positions of most modified residues are highly conserved from yeast to humans and occur in, or in close proximity to functionally important sites, such as the decoding site (DCS), the peptidyl transferase centre (PTC) or the subunit interface (8–11). Prokaryotes and eukaryotes share the majority of modifications in the inner core of the ribosome, while eukaryotes have evolved an additional shell of modifications around the centre of ribosomes (11). This enlarged complexity suggests that eukaryotes developed mechanisms for fine-tuning protein synthesis at the level of rRNA modifications. Changes of those, together with stoichiometric alterations of the protein component and post-translational modifications have led to the ‘specialized ribosome’ concept of translational control (6,7).

We recently reported that lack of a single, conserved C^5^-methylation at 25S rRNA residue C2278 (yeast nomenclature) alters ribosomal structure and translational fidelity, resulting in a ‘reprogramming’ of the ribosome towards translation of mRNAs involved in cellular stress-responses. Importantly, lack of this methylation by deletion of homologs of human NSUN5 extended the lifespan and stress resistance of yeast, worms and flies (12). NSUN5 is a conserved RNA methyltransferase belonging to the Nop2/SUN domain family. So far the only known substrate of the yeast homolog of NSUN5, Rcm1, is C2278 of 25S rRNA (12–14), while C2381 of 26S rRNA is the substrate of NSUN-5, the *Caenorhabditis elegans* homolog (12) and C3782 of human NSUN5 (15). Furthermore, NSUN5 is among 25 heterozygously deleted genes in the rare developmental disorder Williams Beuren syndrome (WBS; OMIM 194050), which is caused by a microdeletion at chromosome region 7q11.23 (16–18). Since recent studies found cognitive deficits in Nsun5 knockout mice (19–21) and WBS patients show neural abnormalities (22), NSUN5 might contribute to the pathology of WBS. Apart from WBS, NSUN5 is also associated with cancer. Recently, down-regulation of this gene by epigenetic silencing was shown to promote long-term survival of glioma patients and to render gliomas more sensitive to bioactive compounds generating oxidative stress (15).

As removal of a single modification of rRNA can already alter the physiology of simple model organisms and impair cognitive function in mice, it becomes evident that a better understanding of these processes in humans is important. This knowledge might then contribute to the development of strategies for improving health of aged subjects, WBS and glioma patients, as well as to further decipher the impact of rRNA modifications on normal physiology.

In this study, we report that loss of NSUN5 decreases growth, cell size and proliferation, as well as bulk protein translation. Moreover, we characterize human NSUN5 regarding its methylation target site, catalytical function, and cellular localization.

## MATERIALS AND METHODS

Methods referring to Supplementary Figures S1–S8, as well as detailed protocols for some of the experimental procedures listed here are provided as supplementary material.

### Cells, animals and ethics

HeLa and HEK293 cells were purchased from ATCC. HeLa cells were cultured in RPMI (F1215, Biochrom) and HEK293 cells in DMEM (F0435, Biochrom), each supplemented with 10% fetal calf serum (FCS) and 4 mM L-glutamine (G7513; Sigma).

Human dermal fibroblasts (HDF) from healthy female donors were obtained from Evercyte GmbH (Vienna, Austria). The two dermal fibroblast strains from Williams Beuren Syndrome (WBS) patients were a kind gift from Dr. Aleksander Hinek (The Hospital for Sick Children, Toronto, Canada). WBSCR14073 was isolated from a skin biopsy of a 6 years old male patient and WBSCR14966 from a skin biopsy of a 3 months old female patient as described previously. Both strains show haploinsufficiency of the NSUN5 gene (23). All human dermal fibroblast strains were cultured in DMEM/Ham's F-12 (1:1 mixture) (F4815, Biochrom) supplemented with 10% FCS and 4 mM L-glutamine (G7513; Sigma). All cell lines and strains were tested for the absence of mycoplasms regularly and kept at 37°C, 7% CO_2_ and 90% humidity.

NSUN5 knockout mice were generated by Applied Stem Cell Inc. by disruption of exon 1 and intron 1 with two loxP-sites by CRISPR-Cas9. Mice were crossed into the C57BL/6J background 10 times. Experiments with Nsun5 knockout mice were approved by the animal ethics commission of the Medical University of Vienna and by the Austrian Federal Ministry of Education, Science and Research (BMBWF-66.009/0199-V/3b/2018). The generation of anti-NSUN5 antibodies was approved by the Austrian Federal Ministry of Science and Research (BMWF-66.016/6-II/3b/2011).

### Isolation and immortalization of mouse embryonic fibroblasts

Embryos from homozygous mating were isolated between E 12.5 and E 14.5. After removing the heads, embryos were minced and incubated with trypsin-EDTA (0.25%) at 37°C for 30 min. Mouse embryonic fibroblast (MEF) cell lines were generated according to (24) and cultured in DMEM (F0435, Biochrom), supplemented with 10% FCS, 4 mM L-glutamine and 0.1 mM β-mercaptoethanol.

### Antibodies

The following primary and secondary antibodies were used for western blot (WB) and immunofluorescence (IF) at indicated dilutions:

**Table 1:** 

Target	Manufacturer	Product #	Host	Conjugate	WB	IF
NSUN5	Santa Cruz	sc-376147	Mouse	–	1:250	1:100
NSUN5	selfmade	–	Mouse	–	1:2000	1:250
β-Actin	Sigma	A-5441	Mouse	–	1:5000	–
GAPDH	Thermo Fisher Scientific	MA5-15738	Mouse	–	1:5000	–
Fibrillarin	Abcam	ab5821	Rabbit	–	–	1: 1000
KRAS, HRAS and NRAS	Abcam	ab55193	Mouse	–	1:1000	–
GFP	Abcam	ab290	Rabbit	–	1:2500	
Mouse IgG	Jackson Immunoresearch	115-485-146	Goat	Dyelight 488	–	1: 1000
Rabbit IgG	Jackson Immunoresearch	111-485-144	Goat	Dyelight 488	–	1: 1000
Rabbit IgG	Jackson Immunoresearch	111-505-144	Goat	Dyelight 549	–	1: 1000
Rabbit IgG	Jackson Immunoresearch	111-495-144	Goat	Dyelight 649	–	1: 1000
Mouse IgG	Life Technologies	O6380	Goat	Oregon Green 488	–	1: 1000
Rabbit IgG	Jackson Immunoresearch	111-065-144	Goat	Biotin	–	1: 1000
Mouse IgG	Life Technologies	A21057	Goat	Alexa Fluor 680	1:10 000	–
Rabbit IgG	Licor	926-32211	Goat	IRDye 800CW	1:10 000	–

For the generation of the polyclonal mouse NSUN5 antibody, recombinant HIS-tagged NSUN5 was expressed in *Escherichia coli* in the pET30a vector and purified using Ni-NTA Agarose beads (Qiagen) under denaturing conditions following the manufacturer's protocol. Recombinant HIS-NSUN5 was used for the immunization of mice. Crude serum from a single mouse was used. A characterization of this antibody is shown in Supplementary Figure S8.

### Generation of NSUN5 KO HeLa cells by CRISPR–Cas9

After reaching 80% confluence, HeLa cells were transfected with an all-in-one plasmid encoding the guide RNA (CTTCACGTTCTGTGTGGCCG, designed by gUIDEbook™), as well as Cas9 and GFP (Horizon Discovery Ltd, Free CRISPR Knockout Generation Program). One day after transfection, 10^5^ GFP-positive cells were isolated with a MoFlo Astrios cell sorter (Beckman Coulter) into a 12-well cell culture plate. On the next day, we performed single cell dilution in 96-well cell culture plates. Clones originating from single cells were screened for NSUN5 expression using western blot and immunofluorescence with the self-made polyclonal NSUN5 mouse antibody.

### Multiple protein sequence alignment of Rcm1p and NSUN family members

A multiple protein sequence alignment of Rcm1p (UniProtKB: P53972), NSUN1 (UniProtKB: P46087), NSUN2 (UniProtKB: Q08J23), NSUN3 (UniProtKB: Q9H649), NSUN4 (UniProtKB: Q96CB9), NSUN5 (UniProtKB: B2RD09), NSUN6 (UniProtKB: Q8TEA1) and NSUN7 (UniProtKB: Q8NE18) was performed with T-Coffee Version_11.00.8cbe486 (25). Clustal colouring was applied. From this alignment a phylogenetic tree with average distances using BLOSSUM62 was constructed in JalView 2.10.5 (26).

### Cell number and cell size determination

Viable cell number and cell size were determined by ViCell XR (Beckmann Coulter). 0.5−1 × 10^5^ cells per well were seeded in 6-well culture plates and total viable cell numbers were determined on consecutive days until 100% confluency was reached. Microscopic images were acquired with an inverted microscope (Leica, DM IL LED) using a 10×/NA 0.22 objective and phase contrast. Insertion of scale bars, as well as brightness and contrast adjustments were done with ImageJ (Version 1.52 e).

### Weight and body composition measurement

Mice of the same litter were weighed at either 4 or 8 weeks of age. Weight ratio per litter was calculated according to this formula, with 2–4 mice per litter:

$$\begin{equation*}{\rm weight}{\rm{\ }}{{\rm ratio}} = \frac{{{{\rm mean}}{\rm{\ }}{{\rm weight}}{\rm{\ }}{{\rm Nsun}}{5^{ - / - }}{\rm{\ }}{{\rm animals}}{\rm{\ }}\left[ {\rm g} \right]}}{{{{\rm mean}}{\rm{\ }}{{\rm weight}}{\rm{\ }}{{\rm Nsun}}{5^{ + / + }}{\rm{\ }}{{\rm animals}}{\rm{\ }}\left[ {\rm g} \right]}}{\rm{\ }} \end{equation*}$$

Body composition of awake un-anesthetized male and female mice between 14 and 18 weeks of age was analysed using EchoMRI-100H (EchoMRI LCC) in a non-invasive manner. The weight of these mice was also recorded.

### Food intake

Male mice between 15 and 19 weeks of age were caged separately. After an adaption period of one week, food was weighed on four consecutive days and the mean food intake of each mouse was calculated.

### Generation and stable transfection of GFP-mNsun5 BACmid

GFP-mNsun5 BACmid was generated by fusing mouse Nsun5 contained in a BAC-clone to the C-terminus of EGFP using the NFLAP tagging cassette (27) by the Quick & Easy BAC Modification Kit (GeneBridges). The GFP-mNsun5 BACmid was amplified in *E. coli* and purified using the Large Construct Purification Kit (Qiagen) following the manufacturer′s instructions. Transfection into HeLa cells was performed using Metafectene Pro (Biontex GmbH) following the manufacturer's instructions. Three days after transfection, 800 μg/ml G418 were applied for selection. Single colonies emerged two weeks after transfection and were screened for GFP-mNsun5 expression and correct intracellular localization by western blot and fluorescence microscopy.

### Site directed mutagenesis of NSUN5 in pCI-neo and lentiviral vectors

The QuickChange II Site-Directed Mutagenesis Kit (Agilent) was applied for exchanging C308 and C359 of NSUN5 in pCI-neo to serines following the manufacturer's instructions. For producing lentiviral particles, the cDNA sequence of wildtype NSUN5 and the two point mutants, C308S and C359S, were cloned into the pLVX-IRES-Hyg expression plasmid (Takara) using In-Fusion^®^ HD Cloning (Takara) with a sense (5′- CGGTGAATTCCTCGAATGGGGCTGTATGCTGCAG-3′) and an antisense (5′-TAGAACTAGTCTCGACTATGTGCAAGGCGGTGTGC-3′) primer at the XhoI restriction site.

### Generation of NSUN5-GFP truncation mutants

The following full-length and truncated versions of NSUN5 were generated by PCR from HeLa cDNA (numbers represent amino acids counted from the N-terminus of the protein, bold letters indicate restriction sites): NSUN5(1–466)-GFP (5′-gataCTCGAGatggggctgtatgctgcagctgc-3′ and 5′-ttagGAATTCatgtgcaaggcggtgtgcaag-3′), NSUN5(1–121)-GFP (5′-gataCTCGAGatggggctgtatgctgcagctgc-3′ and 5′-ttagGAATTCccaggtcctcattccggctcacac-3′), NSUN5(1-277)-GFP (5′-gataCTCGAGatggggctgtatgctgcagctgc-3′ and 5′-ttagGAATTCaagagacgccagcccgggcca-3′), NSUN5(1–428)-GFP (5′-gataCTCGAGatggggctgtatgctgcagctgc-3′ and 5′-ttagGAATTCatggcacctcgacccgttcaatta-3′), NSUN5(121–466)-GFP (5′-gataCTCGAGatgttggaagtgggatccaggcctg-3′ and 5′-ttagGAATTCatgtgcaaggcggtgtgcaag-3′), NSUN5(277–466)-GFP (5′-gataCTCGAGatgtgctgtgaactggctgaggagg-3′ and 5′-ttagGAATTCatgtgcaaggcggtgtgcaag-3′). Purified PCR products were cloned into pEGFP-N1 by restriction digest with XhoI and EcoRI.

### Transfection of mammalian cells

Cells were grown to ∼80% confluence in six-well cell culture plates and transfected according the manufacturer's instructions (jetPRIME Polyplus). Lentiviruses containing NSUN5, C308S and C359S were produced by co-transfecting Lenti-X HEK-293T cells with the recombinant pLVX-IRES-Hyg plasmid and a Lenti-X Packaging Single Shot (Takara) following the supplier's instructions. One day after infection of NSUN5 KO HeLa and MEF −/− cells with virus particles, antibiotic selection (400 μg/ml hygromycin) was initiated. After 5 days of selection, stably expressing cells were expanded and passaged at least three times in media containing 200 μg/ml hygromycin before starting experiments.

### RNA isolation and cDNA synthesis

Cells were lysed in TRI Reagent (Sigma) and RNA was isolated following the manufacturer's protocol. Total RNA was isolated with Direct-zol™ RNA Kit (Zymo Research). RNA quality and concentration were quantified with an ND-1000 (NanoDrop) spectrometer. cDNA was synthesized from 500 ng of total RNA using High-Capacity cDNA Reverse Transcription Kit (Life Technologies).

### RT-qPCR

Target gene expression levels were quantified from cDNA using the 5× HOT FIREPol^®^ EvaGreen^®^ qPCR Mix Plus (Medibena) on a Rotor-Gene Q cycler (Qiagen) with gene-specific primers: GAPDH (human): 5′-CGACCACTTTGTCAAGCTCA-3′ and 5′-TGTGAGGAGGGGAGATTCAG-3′, Actb (mouse): 5′-AGAGGGAAATCGTGCGTGAC-3′ and 5′-CAATAGTGATGACCTGGCCGT-3′, NSUN5 (human): 5′-CTACCATGAGGTCCACTACAT-3′ and 5′-CTGGCAGAGGGAGCA-3′, Nsun5 (mouse): 5′-TTGCAAGAGAGCTCCAGACC-3′ and 5′-AGGCAGCAAGGGATCCAAAA-3′.

### Western blots

Cells and mouse kidneys were lysed in RIPA-buffer (150 mM NaCl, 1% NP-40, 0.5% sodium deoxycholate, 0.1% SDS, 50 mM Tris/HCl pH 8.0), sonicated for 30 cycles (30 s on/30 s off) with a Bioruptor Plus sonicator (Diagenode), centrifuged briefly and mixed with SDS-PAGE sample buffer (60 μM Tris/HCl pH 6.8, 2% SDS, 10% glycerol, 0.0125% bromophenol blue and 1.25% β-mercaptoethanol). Electrophoresis was performed using 4–15% Mini-PROTEAN^®^ TGX Gels (BioRad) in Laemmli-Buffer (25 mM Tris, 250 mM glycine and 0.1% SDS) (28). Protein bands were transferred to PVDF-membranes (Bio Rad) at 25 V and 1.3 A for 3 min. After blocking and antibody incubations, detection was performed on the Odyssey Infrared Imager (LI-COR).

### Immunofluorescence staining of cells for gSTED

Cells were seeded onto coverslips, fixed in 4% formaldehyde and permeabilized in 1% Triton X-100. After incubation with primary antibodies, cells were stained with Oregon Green 488 anti-mouse and biotin anti-rabbit antibodies. Thereafter, coverslips were incubated with V500-streptavidin (Becton Dickinson) and mounted with Mowiol (Sigma). Imaging was performed on a SP8 confocal microscope equipped with a pulsed white-light laser and gSTED (Leica Microsystems). Deconvolution of images was performed with Huygens Professional (Scientific Volume Imaging). Cropping, insertion of scale bars and brightness and contrast adjustments were done with Image J (Version 1.52 e).

### Actinomycin D and α-amanitin exposure

Actinomycin D and α-amanitin treatment was performed as previously described (29). HeLa cells were seeded into μ-slides (ibidi GmbH) and incubated over night at 37°C. On the next day, cells were incubated with 50 ng/ml Actinomycin D (Sigma) or with 50 μg/ml α-amanitin (Sigma) for 2 h. Immunofluorescence staining for NSUN5 (selfmade polyclonal mouse NSUN5 antibody) was performed as described above.

### Bisulfite conversion, PCR amplification and sequencing

Bisulfite conversion of 750 ng DNAse I (Zymo Research) digested total RNA was performed using the EZ RNA Methylation™ Kit (Zymo Research). The converted RNA was eluted in 15 μl nuclease-free water and reverse-transcribed as described above. cDNA after bisulfite conversion was amplified by PCR using GoTaq^®^ DNA polymerase (Promega), forward primer (5′-GGGAGTAATTATGATTTTGACAAGGTAG-3′) and reverse primer (5′-ATAATAAATAAAAACAATAAAAATCTCATTCATCCATTCATACAC-3′) to generate a 101 bp PCR product. After purification by agarose gel electrophoresis and QIAquick PCR Purification Kit (Qiagen), DNA was either used for COBRA assay (see below) or subjected to TOPO cloning using the TOPO TA Cloning Kit for Sequencing (Life Technologies) following the manufacturer's protocol. Purified plasmids of the indicated number of clones were analysed by Sanger sequencing (Eurofins Genomics).

### Combined bisulfite restriction analysis (COBRA)

COBRA was performed as previously described (13) with minor modifications: The PCR product after bisulfite conversion and reverse transcription was digested with MseI (New England Biolabs), resulting in either two products of 45 and 56 bp (methylated) or three bands at 16, 29 and 56 bp (non-methylated). Digested DNA samples were separated on a 20% TBE gel (Life Technologies) followed by incubation for 20 min with SYBR Safe (Life Technologies). The mean density of individual bands was quantified in Image J (Version 1.52 e) and relative methylation [%] was calculated according to this formula:

$$\begin{eqnarray*} &&\!\!\!{{\rm relative}}{\rm{\ }}{{\rm methylation}}{\rm{\ }}\left[ {\rm{\% }} \right] \\ &&\!\!\!= \frac{{{{\rm methylated}}{\rm{\ }}\left( {45{\rm{\ }}{\rm bp}{\rm{\ }}{{\rm band}}} \right)}}{{{{\rm methylated}}{\rm{\ }}\left( {45{\rm{\ }}{\rm bp}{\rm{\ }}{{\rm band}}} \right) + {{\rm non}}{\rm{\ }}{{\rm methylated}}\left( {29{\rm{\ }}{\rm bp}{\rm{\ }}{{\rm band}}} \right)}}{\rm{\times}}100\end{eqnarray*}$$

### *O*-Propargyl-puromycin (OPP) assay

OPP assays were performed as described previously (30). Two specificity controls were included: (i) cells not labelled with OPP and (ii) cells incubated with cycloheximide (#C7698, Sigma) at a final concentration of 50 μg/ml for 15 min prior to, as well as during incubation with OPP.

For quantitation of OPP labeling, cells were analysed by flow cytometry. Selection of whole single cells in G1 phase was based on forward and side scatter, as well as on DAPI staining. Analysis of fluorescence intensities was performed in Kaluza 1.2 (Beckman Coulter).

### OPP labeling *in vivo*

Mice at the age of 4–6 months were weighed and OPP was injected intraperitoneally at a concentration of 49.5 mg/kg of body weight (10 mM OPP in PBS, 10 μl/g). Vehicle control mice were injected with the appropriate amount of PBS. For these experiments, male mice were used, except for four female vehicle control mice. One hour after injection, mice were sacrificed by cervical dislocation. Bone marrow cells were obtained by flushing the femur and tibia with PBS/EtOH (70%). Single cell suspensions of liver, kidney and lung were prepared by washing the tissues in PBS, followed by cutting and incubation with collagenase NB4 (17454, Serva) in DMEM/Ham's F12 (1.7 mg/ml) at 37°C for 30 min. The suspension was pushed through a G23 needle for several times into a petri dish and filtered through a cell strainer (40 μm). After centrifugation, cells were incubated with DNase I in DMEM/Ham's F12 (5 U/ml) at room temperature for 10 min. Cells were harvested by centrifugation, fixed in 1 ml cold EtOH (70%) and stored at 4°C. For visualization of OPP incorporation, cells were treated as described above. For improved comparison of the two independent experiments, OPP labelled samples were normalized to the mean of the respective PBS controls.

### Polysome profiling

Cells were grown to ∼80% confluence and treated with 100 μg/ml cycloheximide for 10 min at 37°C. Subsequently, cells were washed twice with ice-cold PBS containing 100 μg/ml cycloheximide and then resuspended in lysis buffer (30 mM HEPES–KOH pH 7.6, 150 mM KOAc, 5 mM MgOAc_2_, 4 mM DTT, RiboLock RNase Inhibitor (ThermoFisher Scientific), 1× cOmplete™ Protease Inhibitor Cocktail (Roche), 100 μg/ml cycloheximide). Cell membranes were disrupted by passing through a 23G needle for 25 times. Cell debris was removed by centrifugation at ∼15000 g for 15 min and the resulting cleared cell lysate was layered on a linear 10–50% (w/v) sucrose gradient prepared in buffer containing 30 mM HEPES–KOH pH 7.6, 150 mM KOAc, 5 mM MgOAc_2_, 4 mM DTT, 1 mM PMSF, 100 μg/ml cycloheximide and centrifuged for 2 h 45 min at 39 000 rpm in a Beckman SW41 rotor at 4°C. The gradient was then fractionated using a Brandel density gradient fractionation system. Polysome profiles were generated by continuous measurement of the absorbance at 254 nm. Polysome profiles within one biological replicate were normalized to the same square area under the entire profile and the peak area ratio between control HeLa and NSUN5 KO was subsequently calculated.

### *In vitro* translation assay

Cells were grown to ∼80% confluence, washed twice with cold PBS and resuspended in buffer containing 30 mM HEPES–KOH pH 7.6, 150 mM KOAc, 5 mM MgOAc_2_, 4 mM DTT, RiboLock RNase Inhibitor (ThermoFisher Scientific) and 1× cOmplete™ Protease Inhibitor Cocktail (Roche). Cell debris was removed by centrifugation at ∼15 000 g for 15 min and the resulting cleared cell lysate was layered on a 0.5 ml sucrose cushion prepared in 30 mM HEPES–KOH pH 7.6, 150 mM KOAc, 5 mM MgOAc_2_ and centrifuged in a Beckman mini-ultracentrifuge for 2 h 30 min at 200 000 g in a S140AT rotor at 4°C. The pellet (P100 – ribosome fraction) was re-suspended in HEPES–KOH pH 7.6, 150 mM KOAc and 5 mM MgOAc_2_ and the supernatant (S100) was stored on ice. Ribosome content was estimated based on absorbance at 260 nm.

For *in vitro* translation 6.45 pmol of control HeLa or NSUN5 KO HeLa P100 were mixed with 5 μl HeLa S100 in a total volume of 20 μl and pre-incubated for 10 min at 35°C and 450 rpm. During this period, the majority of ribosomes that were already associated with mRNAs will terminate translation. After pre-incubation, 2.1 μl of translation mixture containing 1.2 μl 10× translation cocktail (150 mM HEPES–KOH pH 7.6, 750 mM KOAc, 19.5 mM MgOAc_2_, 4 mM GTP, 17.5 mM ATP, 500 μM 19 amino acids except methionine), 0.17 μl 100 mg/ml bulk yeast tRNA, 0.08 μl 3M creatine phosphate, 0.06 μl 20 mg/ml creatine phosphokinase and 0.625 μl ^35^S-methionine) were added to the reaction (31). *In vitro* translation was performed at 35°C and rotation at 450 rpm for 30 min, stopped by the addition of 8 μl of 4× Laemmli buffer and incubation at 95°C for 5 min. Proteins were separated by 10% SDS PAGE and visualized by Coomassie staining and ^35^S-methionine incorporation by autoradiography.

### Statistics

Statistical analysis and plotting of results were done in R, version 3.5.1 (2018‐07‐02) [R Core Team (2017) R: A Language and Environment for Statistical Computing. Vienna, Austria: R Foundation for Statistical Computing. Retrieved from http://www.R-project.org]. Depending on the shape of the sample distribution, appropriate parametric or non-parametric tests were used as indicated in the figure legend. The number of replicates usually refers to independent biological replicates unless indicated otherwise. For bootstrapping 95% confidence intervals for OPP-Assay, 1000 pseudosamples of equal size to the original dataset were generated by random resampling with replacement. The difference in medians of each pseudosample (in regards to the respective control) was calculated and the 2.5th and 97.5th percentile of the sorted differences in medians were determined as the upper and lower confidence bounds of the CI.

## RESULTS

### NSUN5 is the human homolog of Rcm1p

**Figure 1.**
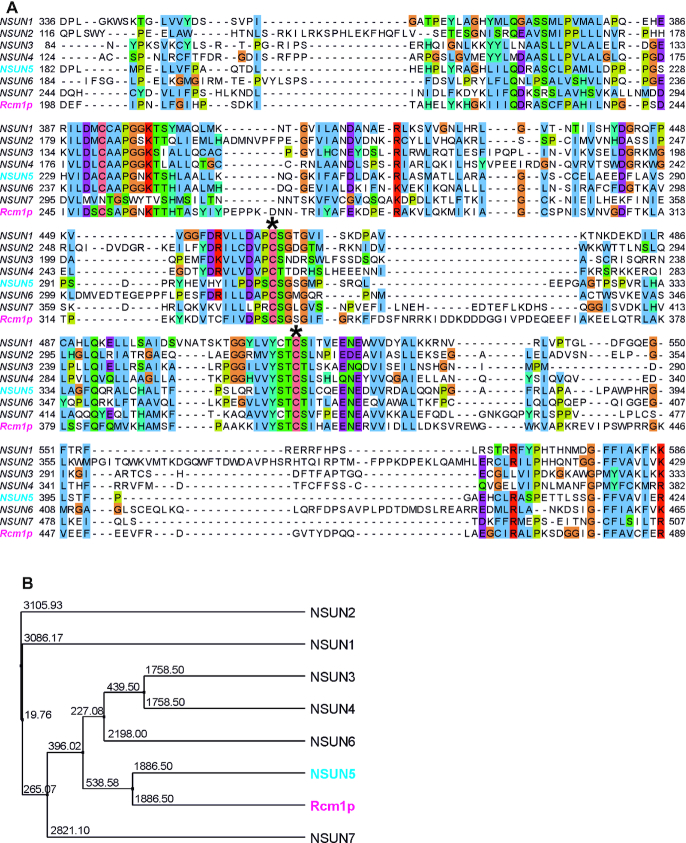
NSUN5 is the closest human homolog of Rcm1p. (**A**) The multiple protein sequence alignment of Rcm1p and the human NSUN protein family is shown. The sequences were cropped as follows: NSUN1 (336-586), NSUN2 (116–429), NSUN3 (84–333), NSUN4 (124–382), NSUN5 (182–424), NSUN6 (184–465), NSUN7 (244–507) and Rcm1p (198–489). The full-length alignment is shown in Supplementary Figure S1. Colors indicate similarities. Two highly conserved cysteins required for covalent RNA substrate binding and subsequent release are indicated by asterisks (*). (**B**) Based on this alignment, the depicted phylogenetic tree was constructed. Numbers indicate phylogenetic distances. NSUN5 (cyan) is the closest human homolog of yeast Rcm1p (magenta).

We previously found that depletion of Rcm1p/NSUN-5/Nsun5 extends lifespan in the model organisms *Saccharomyces cerevisiae*, *Caenorhabditis elegans* and *Drosophila melanogaster* (12). To further investigate the underlying molecular functions and their conservation in mammals, we aligned the protein sequence of Rcm1p from *S. cerevisiae* to all seven human NSUN-family members (Figure 1A and Supplementary Figure S1) to identify a putative mammalian homolog. Evolutionary conservation was highest in the catalytic region, which contains the S-adenosyl methionine (SAM)-binding pocket and cysteines at positions C308 and C359, which are considered to be required for covalent binding and release of the RNA substrate in other RNA methyltransferases (12,32,33). Subsequent phylogenetic analysis suggested that Rcm1p is closest related to human NSUN5 (Figure 1B), which we therefore decided to investigate further.

### Loss of NSUN5 impairs growth of cells and mice

**Figure 2.**
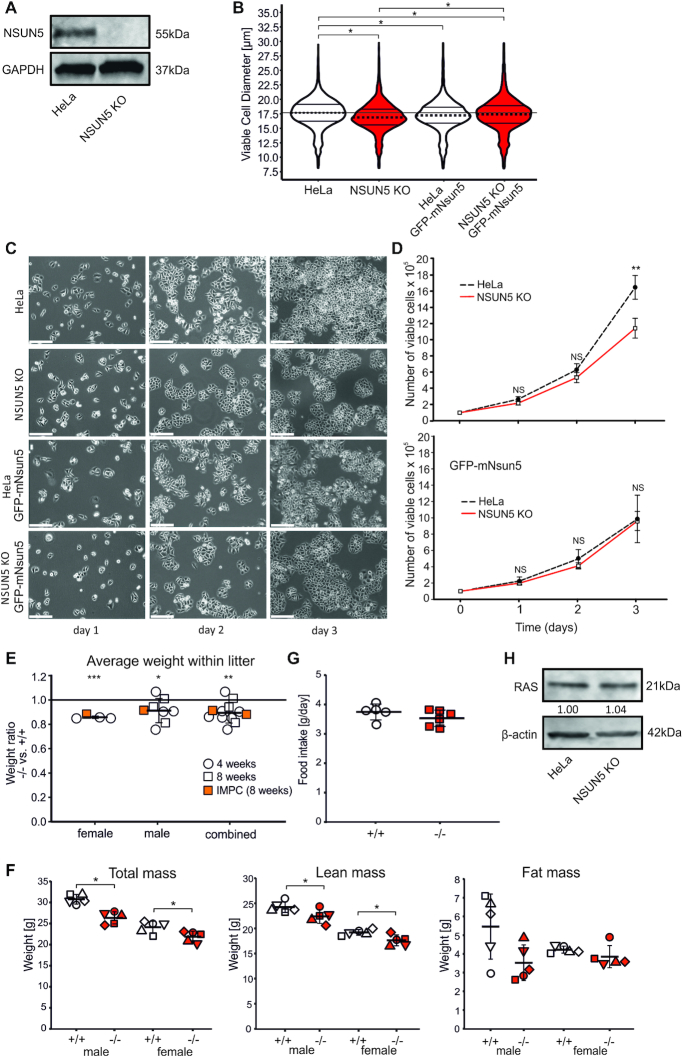
NSUN5 depletion decreases cell size and proliferation without affecting NRAS expression. (**A**) Western blot confirms loss of NSUN5 protein expression in NSUN5 KO compared to HeLa cells. (**B**) Cell size was decreased in NSUN5 KO compared to HeLa cells. Horizontal lines within the violin indicate median, first and third quartile. *n* = 4 independent experiments with ≥6000 cells. *P ≤ 0.05, Kruskal–Wallis rank sum test with Dunn's post-hoc test. (**C**) HeLa, NSUN5 KO, HeLa GFP-mNsun5 and NSUN5 KO GFP-mNsun5 cells on days 1, 2 and 3 after seeding. Representative images of four independent experiments are shown. Scale bar represents 50 μm. (**D**) Analysis of cell proliferation by automated counting of viable cells. *n* = 4 independent experiments with ≥6000 cells each. **P < 0.005, two-tailed Student's *t*-test. ns = not significant. (**E**) The weight of Nsun5 knockout mice was decreased compared to littermate controls. Each transparent data point represents the weight ratio of animals within a single litter of either 4 (circle) or 8 weeks (square) of age. *n* ≥ 4 litters with 2–4 animals each. Data points represented by orange rectangles correspond to publicly available data of another Nsun5 knockout mouse model by the International Mouse Phenotyping Consortium (IMPC), with two knockout (–/–) versus 135 wildtype female (+/+) and 1 knockout (–/–) versus 150 wildtype (+/+) male mice at 8 weeks of age. Error bars indicate standard deviation. *P < 0.05, **P < 0.005, ***P < 0.0005, one sample *t*-test against expected value of 1. (**F**) Total mass, lean mass and fat mass of adult mice at the age of 14–18 weeks assessed by Echo-MRI. Total and lean mass were significantly decreased in Nsun5 knockout compared to wildtype mice. Fat mass revealed no significant differences upon Nsun5 knockout. Shapes of data points always refer to the same individual mouse per group. *n* = 5 per genotype and sex. Error bars indicate standard deviation. *P < 0.05, Welch Two Sample *t*-test. (**G**) Food intake of male mice at the age between 15 and 19 weeks was calculated as mean of four consecutive days. No difference between wildtype (+/+) and Nsun5 knockout (–/–) mice was observed. *n* = 5 (+/+), *n* = 6 (–/–). Welch Two Sample *t*-test. (**H**) Protein expression levels of RAS and β-Actin as loading control were analysed in HeLa and NSUN5 KO cells by western blot. Anti-Ras antibody reacts with NRAS, VRAS and HRAS. Numbers represent quantitation of RAS expression relative to β-Actin. The experiment was independently repeated two times with similar outcome.

To study functional consequences of NSUN5 depletion in a human cell model, we applied CRISPR–Cas9 to homozygously delete NSUN5 in HeLa cells. In order to prevent transcription of functional mRNA, we decided to target the second exon of the NSUN5 gene and subsequently screened clones originating from single cells for loss of NSUN5. We were indeed able to isolate a single clone which showed complete absence of NSUN5 protein expression (Figure 2A). Sanger sequencing of genomic DNA from this clone revealed a translocation from chromosome 4q into intron 1/exon 2 of the NSUN5 locus (Supplementary Figure S2A). This led to loss of NSUN5 mRNA, as confirmed by RT-qPCR (Supplementary Figure S2B).

During cultivation and expansion of this clone, we noticed that cells appeared smaller and proliferated slower than the parental HeLa cell line. To quantify this phenomenon, we measured >6000 single cells and indeed observed a small but significant decrease of 3.5% in median cell diameter in NSUN5 KO (median = 16.85 µm) compared to control HeLa cells (median = 17.75 µm) (Figure 2B). To exclude the possibility that this reduction of cell size was due to off-target effects of CRISPR–Cas9, we stably introduced a BACmid containing the GFP-tagged mouse Nsun5 gene together with its endogenous promotor (27) into NSUN5 KO (NSUN5 KO GFP-mNsun5) and control HeLa (HeLa GFP-mNsun5) cells. Western blot analysis confirmed expression of GFP-mNsun5 at the correct size (Supplementary Figure S3A), while fluorescence microscopy revealed localization predominantly in the nucleoli and in the nucleoplasm (Supplementary Figure S3B). Indeed, expression of GFP-mNsun5 partially rescued the size defect in NSUN5 KO GFP-mNsun5 (median = 17.45 µm) compared to HeLa GFP-mNsun5 cells (median = 17.45 µm) (Figure 2B). The remaining small but significant difference in size might be due to steric effects caused by fusion to GFP or unexpected differential regulation of the mouse genomic locus contained in the BACmid used by the human transcriptional machinery.

To quantify our observation that loss of NSUN5 also decreases proliferation, we seeded equal numbers of HeLa, NSUN5 KO, HeLa GFP-mNsun5 and NSUN5 KO GFP-mNsun5 cells and recorded cell numbers every day. Indeed, HeLa cells reached ∼40% higher cell numbers after three days (Figure 2C), which was also reflected by a steeper growth curve (Figure 2D). Ectopic expression of GFP-mNsun5 slightly suppressed proliferation of HeLa cells, but not of NSUN5 KO cells (Figure 2C, D). To further confirm these results in normal cells, we introduced four different small hairpin RNAs (shRNAs) targeting NSUN5, as well as a non-hairpin forming control, into three different strains of human dermal fibroblasts (HDFs). After puromycin selection, reduced expression of NSUN5 mRNA was confirmed by RT-qPCR (Supplementary Figure S4A) and reduced cell numbers were observed in all fibroblast strains transduced with shRNA constructs compared to the non-hairpin forming control (Supplementary Figure S4B). To exclude the possibility that the reduction of cell numbers was due to acute infection stress, we re-seeded one of the HDF strains at the same cell density and again, a decrease in proliferation upon NSUN5 depletion was evident (Supplementary Figure S4C).

To assess the consequences of Nsun5 loss in mammals *in vivo*, we deleted exon 1 of the Nsun5 gene in C57BL/6J mice by CRISPR/Cas9, which resulted in Nsun5 knockout in the whole mouse body (Supplementary Figure S4D). We confirmed that Nsun5 was indeed not expressed in these mice at mRNA and protein level by RT-qPCR and western blot (Supplementary Figure S4E and F). When recording the weight of Nsun5 knockout and wildtype animals within the same litter at either 4 or 8 weeks of age, we observed a significant decrease in body weight by ∼10% in both sexes upon loss of Nsun5, which was slightly more pronounced in females than in males (Figure 2E). A similar trend was visible in two other Nsun5 knockout mouse models, one by the International Mouse Phenotyping consortium (34) at 8 weeks of age (data included in Figure 2E), as well as in a recent publication by Zhang and coworkers, although not reaching statistical significance (21). Interestingly, also adult Nsun5 knockout mice at 14–18 weeks of age were significantly lighter than wildtype mice (Figure 2F). EchoMRI measurements revealed that the weight difference between wildtype and Nsun5 knockout animals was evident in lean but not in fat mass (Figure 2F). Food intake of Nsun5 knockout mice (mean = 3.53 g/day) was slightly reduced compared to wildtype animals (mean = 3.75 g/day), but did not reach statistical significance (Figure 2G).

Herdy *et al.* found that NSUN5 interacts with the 5′UTR of NRAS mRNA. As RAS represents a proto-oncogene regulating cell growth, we speculated that the growth phenotypes we observed upon NSUN5 depletion might be caused by changes in RAS expression. To test this hypothesis, we measured RAS protein levels in HeLa and NSUN5 KO cells, but did not observe any differences (Figure 2H).

Taken together, these experiments clearly indicate that NSUN5 is partially required for reaching a specific cell size and for proliferation in mammals without influencing RAS protein levels. Moreover, Nsun5 depletion resulted in reduced body weight of mice, prompting us to further investigate the underlying molecular mechanisms.

### NSUN5 methylates C3782 of human and C3438 of mouse 28S ribosomal RNA

**Figure 3.**
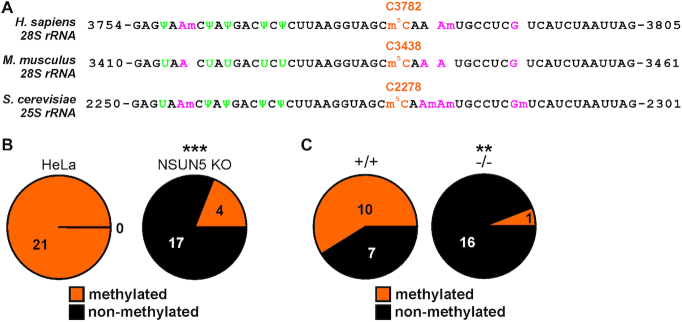
NSUN5 methylates C3782 of 28S rRNA in human and C3438 in murine cells. (**A**) The sequence of 25S/28S ribosomal RNA surrounding the putative methylation site of NSUN5 (orange color) is highly conserved between yeast, mice and humans. 2′-O-methyls are depicted in magenta and pseudouridines (Ψ) in green. (**B**) Bisulfite conversion and Sanger sequencing of RNA from HeLa and NSUN5 KO cells confirms C3782 methylation by NSUN5. Figures indicate numbers of clones with (orange) or without (black) methylation. Pooled results from two independent experiments are shown. Pooled *n* = 21 per condition. ***P < 0.0005, Fisher's exact test for count data. (**C**) Bisulfite conversion and Sanger sequencing of RNA from kidneys of wildtype (+/+) and NSUN5 KO (–/–) mice confirms C3438 methylation by NSUN5. Numbers indicate clones with (orange) or without (black) methylation. Pooled results from two independent experiments of littermate animals are shown. Pooled *n* = 17 per condition. **P < 0.005, Fisher's exact test for count data.

Since we previously did not observe similar size and growth phenotypes in *S. cerevisiae*, *C. elegans* or *D. melanogaster* upon NSUN5 depletion (12), we first aimed to confirm that human NSUN5 methyltransferase activity is indeed directed against a specific cytosine of 28S rRNA, as previously hypothesized in literature (10,11,35) and shown in yeast, worms and human glioma cells (12,14,15). Therefore, we compared the sequences of 28S rRNA of humans and mice with the sequence stretch on 25S rRNA that is methylated by Rcm1 in yeast (Figure 3A). Indeed, we observed 100% evolutionary conservation of the nucleotide sequence, as well as conservation of many other known RNA modifications, indicating functional importance of this region. Due to the high similarity, we hypothesized that NSUN5 might methylate C3782 in humans. To test this hypothesis, we performed bisulfite treatment of total RNA, followed by reverse transcription, PCR amplification, TOPO-cloning and Sanger-sequencing of 21 individual clones from HeLa and NSUN5 KO cells, each. All 21 sequences of normal HeLa cells showed no conversion of C3782 to uridine, indicating that this residue of 28S rRNA was fully methylated. In contrast, only 4 of 21 (19%) clones of NSUN5 KO cells were not converted (Figure 3B). Thus, NSUN5 is clearly responsible for m^5^C modification of C3782 of 28S rRNA. Our findings are corroborated by another recent study, showing that C3782 was the only methylation site altered upon ectopic expression of NSUN5 in a NSUN5 lacking glioma cell line (15).

Due to the high evolutionary conservation of the sequence stretch of 28S rRNA (Figure 3A), we hypothesized that Nsun5 methylates C3438 on 28S rRNA in mice. We tested this hypothesis by using the same approach as for HeLa cells with kidneys of Nsun5 knockout and littermate control mice. Indeed, more than half of 17 clones of wildtype mice showed methylation at C3438, in contrast to only one single clone of Nsun5 knockout animals (Figure 3C), confirming that Nsun5 is responsible for the modification of C3438 of 28S rRNA in mice.

### Two conserved cysteines of NSUN5 are required for its RNA methyltransferase activity and normal proliferation

**Figure 4.**
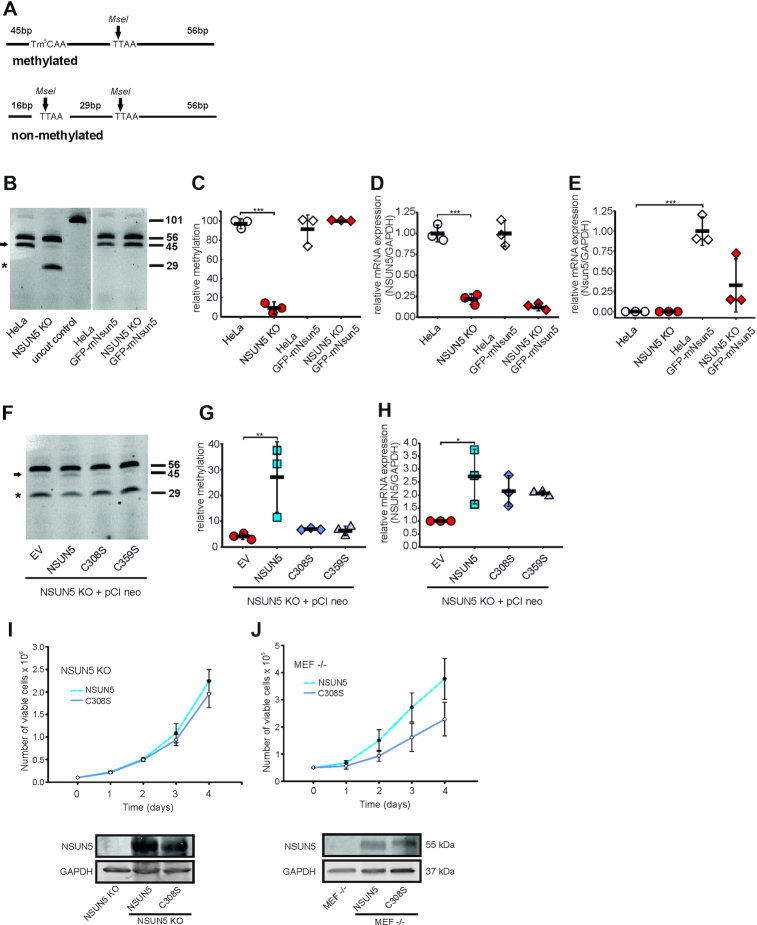
Two conserved cysteines of NSUN5 are required for RNA methyltransferase activity and normal proliferation. (**A**) Principle of the COBRA assay to measure m^5^C3782. Digestion with MseI generates two products (56 bp and 45 bp) in the presence of m^5^C3782 and three products (56, 29 and 16 bp) in the absence of m^5^C3782. A band at 101 bp represents the undigested PCR product. (**B**) COBRA assay of HeLa, NSUN5 KO, HeLa GFP-mNSUN5 and NSUN5 KO GFP-mNSUN5 demonstrates that mouse Nsun5 fully restores methylation of C3782 upon loss of human NSUN5. A representative 20% TBE gel of MseI-digested PCR products is shown. Numbers indicate size of fragments in basepairs. Arrow indicates methylation-specific band, whereas asterisk (*) indicates band upon non-methylation. (**C**) Quantitation of COBRA assay in (B), *n* = 3 independent experiments. Error bars represent standard deviation. ***P < 0.0005, one-way-ANOVA followed by a post-hoc Dunnet's test. (**D**, **E**) mRNA expression levels of human NSUN5 (**D**) and mouse Nsun5 (**E**) were analyzed by RT-qPCR. *n* = 3 independent experiments. Error bars represent standard deviations. ***P < 0.0005, one-way-ANOVA followed by a post-hoc Dunnet's test. (**F**) Representative COBRA assay of NSUN5 KO cells stably expressing the empty pCI-neo vector (EV) or pCI-neo containing either NSUN5, NSUN5 (C308S) or NSUN5 (C359S) demonstrates the requirement of C308S and C359S for catalysis. (**G**) Quantitation of COBRA assay in (F), *n* = 3 independent experiments. Error bars represent standard deviations. **P < 0.005, one-way-ANOVA followed by a post-hoc Dunnet's test. (**H**) Ectopic expression of NSUN5 in pCI-neo restores NSUN5 mRNA levels as analysed by RT-qPCR. *n* = 3 independent experiments. Error bars indicate standard deviations. *P < 0.05, one-way-ANOVA followed by a post-hoc Dunnet's test. (**I**, **J**) Analysis of cell proliferation by automated counting of viable cells comparing NSUN5 KO HeLa (**I**) or MEF –/– (**J**) ectopically expressing either human NSUN5 or the C308S mutant on four consecutive days after seeding. Error bars indicate standard deviations. *n* = 4 independent experiments. Western blots confirm ectopic expression of NSUN5 in both cell types.

In order to confirm this result by a different and faster read-out, we adapted the Combined Bisulfite Restriction Analysis (COBRA) assay for detection of m^5^C2278 in yeast (13) to human cells (Figure 4A). This approach confirmed the results from Sanger-sequencing after bisulfite conversion, also showing low potential residual methylation. To exclude artefacts from using HeLa cells and off-target effects by CRISPR–Cas9 deletion, we knocked down NSUN5 mRNA expression in HEK293 by shRNAs and observed, despite low knockdown efficiency in this cell type (Supplementary Figure S5A), a trend towards loss of m^5^C3782, although not reaching statistical significance (Supplementary Figure S5B).

Due to the high evolutionary conservation of NSUN5 and its rRNA target site between human and mouse, we aimed to test whether mouse Nsun5 can rescue loss of NSUN5 in human cells. Indeed, ectopic expression of endogenous levels of GFP-mNsun5 encoded on the BACmid was able to fully restore methylation of m^5^C3782 in HeLa cells (Figure 4B, C). Loss of human NSUN5 mRNA (Figure 4D) and gain of mouse Nsun5 mRNA (Figure 4E) were confirmed by RT-qPCR.

To identify amino acid residues involved in the catalysis of the methylation, we focused on two cysteine residues of NSUN5 that we considered to be essential in the binding and release of the substrate due to their conserved positioning and amino acid surroundings (Supplementary Figure S1) (12,32,33). We then performed site directed mutagenesis to exchange these cysteines to serines (C308S and C359S). In fact, ectopic overexpression of human NSUN5 rescued C3782 methylation defects as visualized by COBRA assay, while C308S and C359S mutant versions of NSUN5 did not (Figure 4F, G). Restored NSUN5 mRNA expression was confirmed by RT-qPCR (Figure 4H). Moreover, ectopic expression of human wildtype NSUN5 in HeLa and mouse embryonic fibroblast (MEF) cells lacking NSUN5 led to increased proliferation compared to cells ectopically expressing the inactive C308S mutant (Figure 4I, J). We were not able to test the C359S mutation, as several attempts to generate stable cell lines failed. We hypothesize that this might be due to an inability to release covalently bound NSUN5 from the RNA, resulting in impaired rRNA biogenesis and consequently cellular toxicity.

We therefore conclude that NSUN5 is responsible and required for introduction of m^5^C3782 within an evolutionarily highly conserved region of 28S rRNA via two conserved cysteine residues, while loss of this catalytic activity is directly linked to decreased proliferation.

### RNA polymerase I activity and the N-terminal domain are required for nucleolar localization of NSUN5

**Figure 5.**
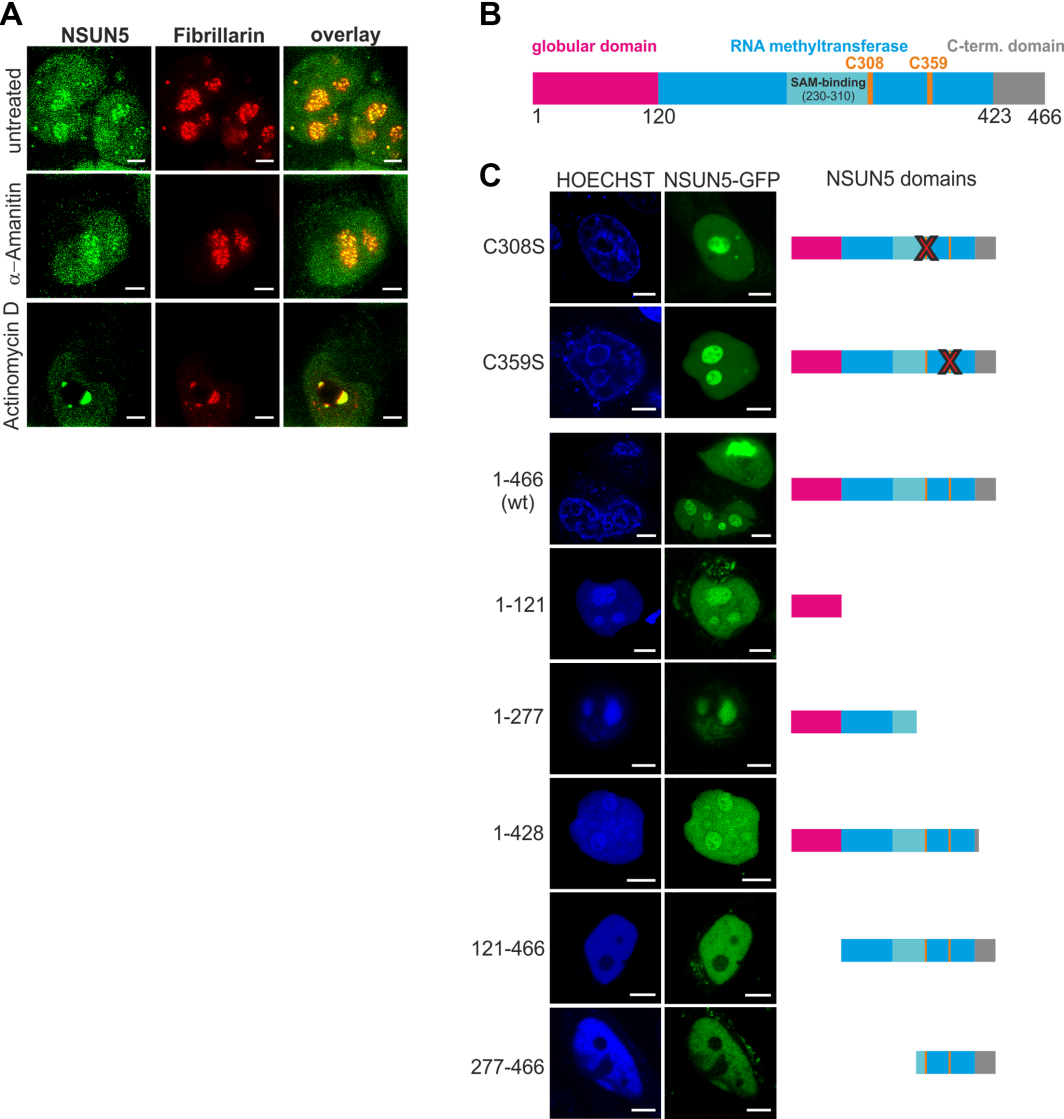
RNA polymerase I activity and presence of the N-terminal domain are required for nucleolar localization of NSUN5. (**A**) HeLa cells were exposed to 50 μg/ml α-amanitin to block RNA Polymerase II and III or 50 ng/ml actinomycin D to block RNA Polymerase I, II and III. NSUN5 (green) and fibrillarin (red) were visualized by indirect immunofluorescence staining. Confocal gSTED microscopy reveals nucleolar localization of NSUN5 and segregation to nucleolar caps upon inhibition of RNA polymerase I. Images were processed by deconvolution and brightness and contrasts were adjusted. Scale bar represents 3 μm. The experiment was repeated twice with similar outcome (see also Supplementary Figure S6). (**B**) Protein domain structure of NSUN5. The N-terminal globular domain (magenta), the RNA methyltransferase domain (blue), the C-terminal domain (grey), the two cysteins required for catalysis (C308 and C359, orange) and the SAM (S-adenosyl methionine) binding site (cyan) are depicted. Numbers indicate amino acids counted from the N-terminus. (**C**) HeLa cells were transfected with NSUN5 (C308S, full-length), NSUN5 (C359S, full-length), NSUN5 (1–466, full-length), NSUN5 (1–121), NSUN5 (1–277), NSUN5 (1–428), NSUN5 (121–466) and NSUN5 (277–466) as C-terminal GFP-fusions. Confocal microscopy revealed nucleolar localization of all NSUN5 constructs (green) except NSUN5 (121–466) and NSUN5 (277–466). Nuclei were counterstained with Hoechst33342 (blue). Microscopic images and NSUN5 domain structure of mutants are shown side by side. Images were processed by deconvolution and brightness and contrasts were adjusted. Scale bar represents 5 μm. The experiment was repeated twice with similar outcome.

All four eukaryotic rRNAs are transcribed in the nucleolus by RNA polymerases I and III, representing the first steps of ribosome biogenesis. Consequently, the pre-ribosomal particles are exported to the cytoplasm, where mature ribosomes are assembled (36–38). To test in which cellular compartment m^5^C3782 is introduced by NSUN5, we performed immunofluorescence staining of HeLa cells and analysed endogenous NSUN5 localization by gated STED super resolution microscopy. Nucleoli were stained with an antibody targeting fibrillarin (Figure 5A, Supplementary Figure S6). As expected and also observed for GFP-mNsun5 (Supplementary Figure S3B), NSUN5 was present in the centre of nucleoli, which suggests that m^5^C3782 is introduced at an early stage of ribosome biogenesis. Next, we specifically blocked RNA polymerase II and III with α-amanitin, which left the nucleolar localization of NSUN5 and fibrillarin undisturbed (Figure 5A). RNA polymerase II synthesizes mRNAs and microRNAs, while RNA polymerase III transcribes tRNAs and 5S rRNA (36,38). However, when cells were exposed to 50 ng/ml actinomycin D to specifically block RNA polymerase I (39–41) which transcribes the pre-rRNA template that is further processed into 28S, 18S and 5.8S rRNA, both NSUN5 and fibrillarin segregated to the nucleolar caps (Figure 5A), as previously described for other nucleolar proteins as well (42).

Next, we aimed to test which part of NSUN5 is required for nucleolar localization. NSUN5 is composed of three domains: The N-terminal domain of unknown function and predicted globular tertiary structure, the evolutionarily conserved RNA methyltransferase domain including the SAM binding pocket and two cysteines required for catalysis, as well as the C-terminal domain of unknown function (Figure 5B, Supplementary Figure S1). First, we ectopically expressed full-length NSUN5 (NSUN5-GFP), as well as the C308S and C359S mutants, with a C-terminal GFP-tag in normal HeLa cells. Neither C308S nor C359S did affect nucleolar localization (Figure 5C), indicating that the catalytic activity is not required for targeting NSUN5 to its substrate. Thus, we generated truncation mutants of NSUN5-GFP and observed that complete absence of the N-terminal globular domain fully excluded NSUN5-GFP from nucleoli, while absence of the C-terminal domain only led to a slight redistribution from nucleoli towards the nucleoplasm (Figure 5C).

Taken together, these findings suggest that RNA polymerase I activity, as well as presence of the globular N-terminal domain, and to a much lesser extend also of the C-terminal domain, are required for nucleolar localization of NSUN5. Consequently, we suggest that the addition of m^5^C3782 occurs co-transcriptionally and is guided by the globular N-terminal domain of NSUN5, representing the functional targeting moiety.

### Loss of NSUN5 reduces global protein translation but does not affect ribosome biogenesis and fidelity

**Figure 6.**
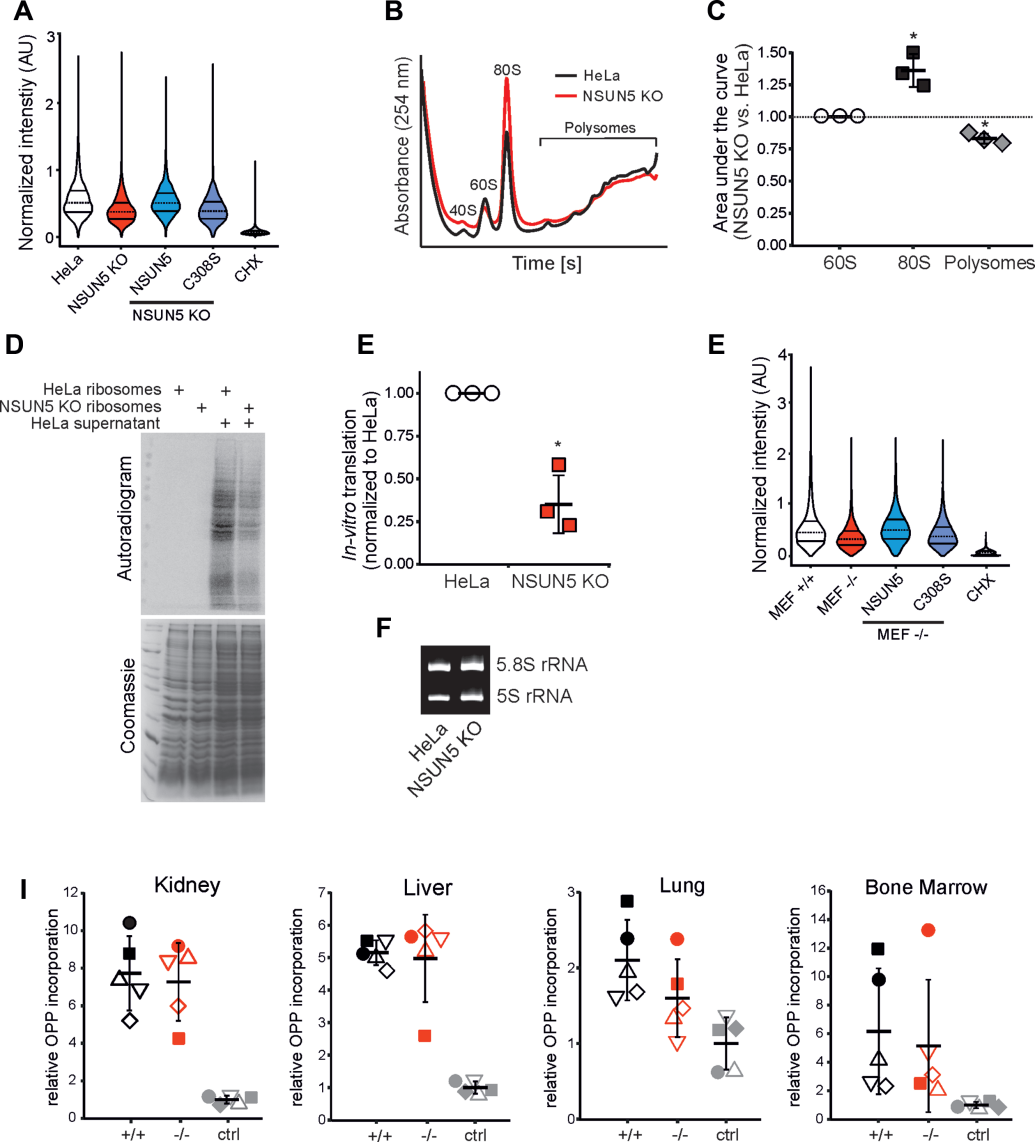
Loss of NSUN5 reduces global protein translation. (**A**) Total protein synthesis, analysed by OPP incorporation and flow cytometry, was reduced in NSUN5 KO compared to control HeLa cells and could be rescued by ectopic expression of NSUN5 but not by the C308S mutant. Specificity was verified by cycloheximide (CHX) exposure or omission of OPP (data not shown). Horizontal lines within the violin represent median, first and third quartile. Pooled data from 4 independent replicates are shown. 5000 cells of each condition were randomly selected for analysis and normalized to the sum of fluorescence intensities of all selected cells within the replicate. Bootstrapped 95% confidence intervals (CI) on difference in medians to HeLa in %: NSUN5 KO = −27.3 to −25.3; NSUN5 KO ectopically expressing NSUN5 = −1.8 to 0.4; NSUN5 KO ectopically expressing C308S = −25.0 to −22.8; CHX = −87.3 to −85.5. (**B**) Loss of NSUN5 increases the 80S monosome peak compared to polysomes in HeLa cells. Polysome profiling was performed three times independently, representative profiles are shown. (**C**) Quantification of polysome profiles by calculating the area under the 60S, 80S and polysome peak, respectively. Data were normalized to 60S. Error bars indicate standard deviation. *P < 0.05, one sample t-test against expected value of 1. (**D**) Ribosomes of control HeLa and NSUN5 KO cells were purified and spiked into ribosome-free supernatant of HeLa cells. Autoradiogram shows decreased amounts of nascent *in vitro* translated proteins upon NSUN5 loss. Purified ribosomes without supernatant verified specificity and Coomassie staining confirmed equal loading. (**E**) Quantitation of autoradiograms demonstrate less *in vitro* translation in NSUN5 KO compared to control HeLa cells. Error bars indicate standard deviation. *P < 0.05, one sample *t*-test against expected value of 1. (**F**) Ethidium bromide staining of a denaturing polyacrylamide gel confirms equal loading of purified ribosomes. (**G**) Total protein synthesis, analysed by OPP incorporation and flow cytometry, of MEF –/– was reduced compared to MEF +/+ cells and could be rescued by ectopic expression of NSUN5 but not by the C308S mutant. Specificity was verified by cycloheximide (CHX) exposure or omission of OPP (data not shown). Horizontal lines within the violin represent median, first and third quartile. Pooled data from three independent replicates are shown. 5000 cells of each condition were randomly selected for analysis and normalized to the sum of fluorescence intensities of all selected cells within the replicate. Bootstrapped 95% confidence intervals (CI) on difference in medians to MEF +/+ in %: MEF –/– = −29.2 to −26.1; MEF -/- ectopically expressing NSUN5 = −8.3 to 11.6; MEF –/– ectopically expressing C308S = −18.7 to −15.5; CHX = −87.1 to −84.7. (**H**) Total protein synthesis *in vivo* was analysed by injection of OPP and flow cytometry of single cell suspensions of kidney, liver, lung and bone marrow. Specificity was verified by injection of PBS (ctrl). *N* = 5 animals per condition in two independent experiments (indicated by either filled or empty symbols). Welch Two Sample *t*-test: Kidney: *P* = 0.7313; mean +/+ = 7.730, mean –/– = 7.274; Liver: *P* = 0.7882, mean +/+ = 5.1526, mean –/– = 4.9744; Lung: *P* = 0.1684, mean +/+ = 2.102, mean –/– = 1.600; bone marrow: *P* = 0.7305, +/+ = 6.162, mean –/– = 5.140.

To further elucidate molecular mechanisms that might explain the observed growth and proliferation phenotype, we aimed to investigate the potential role of m^5^C3782 in global protein translation in HeLa cells. Quantification of O-propargyl-puromycin (OPP) incorporation by flow cytometry (30) revealed median bulk translation to be reduced by about 25% in NSUN5 KO (median = 0.379) compared to control HeLa cells (median = 0.513) (Figure 6A). This reduction in protein synthesis is directly connected with the methylation activity of NSUN5, since ectopic expression of wildtype NSUN5 in NSUN5 KO cells was able to rescue OPP incorporation (median = 0.510), while expression of the catalytically inactive mutant C308S was not (median = 0.391) (Figure 6A). Similarly, comparison of polysome profiles, which provide a snapshot of translational activity, showed an increase of 80S monosomes compared to polysomes upon loss of NSUN5 (Figure 6B, C).

To confirm that alterations to the ribosome itself, and not the number of ribosomes or other extrinsic factors, which might also be influenced by loss of NSUN5, are responsible for decreased bulk protein synthesis, we decided to analyse translation *in vitro*. For this aim, we depleted ribosomes from a HeLa cell lysate by ultracentrifugation and spiked this extract with equal amounts of purified ribosomes from either control HeLa cells or NSUN5 KO cells, as well as with ^35^S-Methionine to quantify protein synthesis. Thereby, we observed a significant reduction in ^35^S-Methionine incorporation upon loss of NSUN5, whereas the size distribution of newly synthesized proteins appeared to be similar (Figure 6D). Omission of the supernatant, which contains several components necessary for translation including mRNA, tRNAs, initiation, elongation and termination factors, did not result in protein synthesis and thereby confirmed specificity of the assay. Quantification of *in vitro* synthesized, radiolabelled protein over the full length of the autoradiogram revealed a reduction by 65% in NSUN5 KO (mean = 0.35) compared to control HeLa cells (mean = 1) (Figure 6E). We confirmed equal loading of extracts by Coomassie staining and equal amounts of ribosomes by ethidium bromide staining for 5S and 5.8S rRNA (Figure 6F).

Importantly, the observed protein synthesis defect in HeLa cells is not associated with decreased amounts of mature ribosomes per cell, as quantified by comparing 28S and 18S bands upon loading of total RNA from equal numbers of HeLa and NSUN5 KO cells (Supplementary Figure S7A). Furthermore, loss of NSUN5 in HeLa cells did not induce any obvious alterations in ribosome biogenesis, as evidenced by northern blot analysis with two specific pre-rRNA probes (Supplementary Figure S7B–E).

Cancer cells, such as HeLa or glioblastoma, and cells of embryonic origin, such as MEFs, require high global protein translation rates to maintain their fast proliferation behavior. For this reason, we decided to measure protein translation also in MEFs, as well as in various normal tissues of wildtype and Nsun5 knockout mice *in vivo* by injection of OPP. OPP labelling of Nsun5 deficient MEFs (median = 0.335) indeed revealed a substantial reduction in overall protein synthesis compared to wildtype control (median = 0.464), which could be rescued by ectopic expression of human NSUN5 (median = 0.509), but not by the C308S mutant (median = 0.384) (Figure 6G). Interestingly, quantification of OPP incorporation into liver, kidney, lung and bone marrow also showed a trend towards decreased protein synthesis in Nsun5 knockout animals compared to littermate controls (Figure 6H), but to a lesser extent then in MEFs.

Since we previously observed that loss of Rcm1, the NSUN5 orthologue in yeast, increased stop-codon read-through (12), and depletion of *nsun-5* in a polyglutamine-frameshifting *C. elegans* model caused elevated -1 translational frameshifting upon osmotic stress (43), we aimed to test if loss of human NSUN5 also influences stop-codon read-through or perturbs translational fidelity in other ways. However, the recognition of any of the three stop-codons was not affected in NSUN5 KO compared to control HeLa cells upon transient transfection of dual luciferase-based reporter constructs (Supplementary Figure S7F). Moreover, neither translational frameshifting induced by viral sequences (44) (Supplementary Figure S7G), nor amino acid misincorporation (45,46) were altered upon NSUN5 loss (Supplementary Figure S7H). Additionally, recognition of internal ribosome entry sites (IRES) (Supplementary Figure S7I), which was previously reported to be affected by pseudouridines in rRNA (47), was not different in NSUN5 KO versus control HeLa cells.

Taken together, our findings indicate that presence of NSUN5-mediated 28S rRNA methylation is required for maintaining productive global protein synthesis, but not translational fidelity, IRES recognition or ribosome biogenesis in mammals.

### Partial loss of NSUN5 in Williams-Beuren-Syndrome is sufficient to reduce m^5^C3782

NSUN5 is deleted in around 95% of WBS patients (48), who show clinical phenotypes including growth retardation, a dysmorphic face, behavioral and cognitive alterations, hypercalcaemia, as well as aortic and pulmonary stenosis (49). While haploinsufficiency of the elastin gene was unambiguously associated with the cardiovascular disease in WBS, the question which other genes are dosage-sensitive and contribute the clinical phenotype still remains unanswered (49). Recently Zhang *et al.* observed cognitive impairments in homozygous Nsun5 knockout mice, which were associated with a decrease in proliferation of oligodendrocyte precursor cells (21).

**Figure 7.**
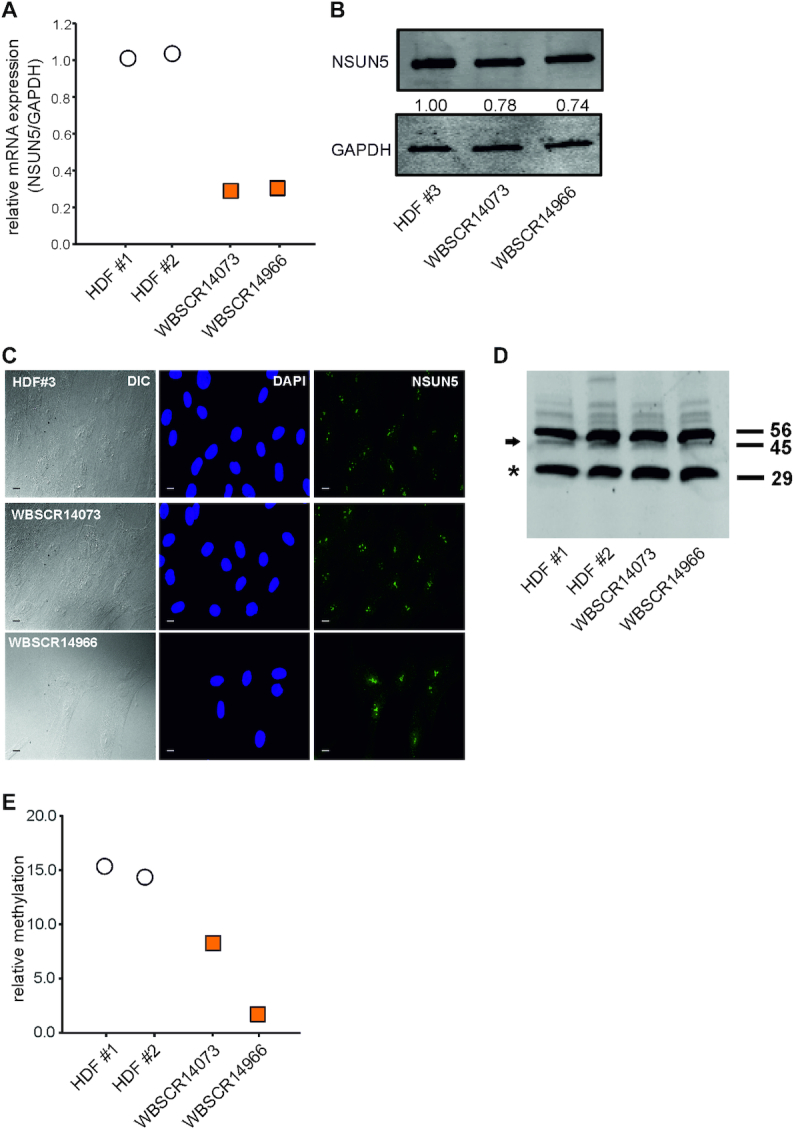
Partial loss of NSUN5 in Williams-Beuren-Syndrome is sufficient to reduce m^5^C3782. (**A**) Relative expression levels of NSUN5 mRNA of two healthy donors as well as two WBS patients were determined by RT-qPCR. (**B**) Protein expression levels of NSUN5 were measured in fibroblasts of a healthy donor and two WBS patients. (**C**) DIC and IF images of fibroblasts of a healthy donor and the two WBS patients. NSUN5 is stained in green and nuclei are counterstained with DAPI. Scale bar represents 10 μm. (**D**) COBRA assay of fibroblasts of two healthy donors and two WBS patients indicates that methylation of C3782 is decreased in WBS. A representative 20% TBE gel of MseI-digested PCR products is shown. Numbers indicate size of fragments in basepairs. Arrow indicates methylation-specific band, whereas asterisk (*) indicates band upon non-methylation. (**E**) Quantitation of COBRA assay in (D).

This prompted us to further investigate the functional role of NSUN5 in WBS. To assess whether haploinsufficiency of the NSUN5 gene affects mRNA expression, we compared NSUN5 mRNA levels in HDF of two WBS patients to two healthy donors. Indeed, we observed reduced levels of NSUN5 mRNA in both cell strains derived from WBS patients (Figure 7A). Similarly, reduced expression of NSUN5 protein was detected by western blot (Figure 7B), while nucleolar localization of endogenous NSUN5 was not altered (Figure 7C). To test whether the partial loss of NSUN5 is also sufficient to reduce methylation of C3782, we performed COBRA assay and indeed observed decreased C3782 methylation in HDF from two WBS patients compared to two healthy donors (Figure 7D, E).

Thus, we conclude that NSUN5 expression is lower in HDF from WBS patients compared to cells from healthy donors on both mRNA and protein level. Furthermore, reduced NSUN5 expression also results in decreased C3782 methylation. However, further studies in heterozygous Nsun5 knockout mice are required to assess whether the reduced gene dosage by Nsun5 haploinsufficiency is enough to induce similar cognitive deficits that were observed in Nsun5 full knockout mice (19–21).

## DISCUSSION

We here report that loss of NSUN5 induces growth phenotypes in mice and different mammalian cells, which is likely connected to the requirement of NSUN5 for ribosomal RNA modification and normal global protein translation, especially in highly proliferative cells. However, we can also not fully rule out that NSUN5 directly disturbs one of the main growth pathways or RAS activity without interfering with RAS protein levels. But how might loss of m^5^C3782 affect the ribosome?

Upon assembly of the SSU and LSU to active 80S ribosomes, the eukaryote-specific bridge eB14 is formed, which directly contacts rRNA base modifications of both subunits including m^5^C3782. This residue, which we have shown to be methylated by NSUN5, is located within helix 70, domain IV on 28S rRNA and interacts with RPL41, a small but highly positively charged protein and main constituent of eB14, which expands from the 60S and protrudes into a binding pocket of the 40S DCS (50). Although not much is known about the presence and precise function of the eB14 bridge in humans (10,11,50), NSUN5-guided methylation of C3782 might play an important part in its formation and thereby modulate global protein translation.

Accumulating evidence for the presence of ‘specialized ribosomes’, which are generated in response to various stimuli and promote the selection of specific mRNAs for translation (6,7), fuels the hypothesis that presence or absence of certain rRNA modifications might contribute to ribosome heterogeneity on a structural and functional level. Our discovery that NSUN5, being required for the addition of a specific methylation to 28S rRNA in humans and mice, co-localizes with fibrillarin in the centre of nucleoli suggests that addition of m^5^C3782 might constitute an early event during ribosome biogenesis happening co-transcriptionally, as was already reported for 2′O-methylations introduced by fibrillarin (51,52). As m^5^C3782 is located in the core of mature ribosomes, this site appears unlikely to be accessible for hypothetical cytoplasmic RNA methylases or de-methylases. Since the response time of transmitting a signal via NSUN5-guided methylation of rRNA in the nucleolus to mature (specialized) ribosomes leading ultimately to altered mRNA translation patterns would be rather slow compared to other cellular regulatory loops, we consider it hardly probable that NSUN5 plays an active role in the translational regulation of gene expression. Nonetheless, the identification of potential upstream regulators of NSUN5 expression and activity is important and will be further studied, as tight regulation of NSUN5 expression levels seems to be crucial for cellular fitness.

Recent studies revealed almost complete methylation at C3782 in HeLa (53) and at C3438 in mouse embryonic stem cells (54). However, as we found only partial methylation in mouse kidney, it will be interesting to determine in future studies if these discrepancies are caused by artefacts due to the much lower sequencing depth of our approach. In addition, methylation levels might be influenced by the tissue context or the proliferative capacity of cells.

Equally important will be the identification of potential additional RNA substrates of NSUN5 by global bisulfite sequencing, Aza-IP or miCLIP (33,53–56), which will also clarify whether the recently described physical association of NSUN5 with NRAS mRNA in cytoplasmic extracts (57) results in NRAS mRNA methylation, or if NSUN5 fulfils other non-canonical functions there. Surprisingly and contradictory to the study by Herdy and coworkers (57), we did not observe cytoplasmic localization of NSUN5, neither with two different antibodies detecting endogenous NSUN5, nor by ectopic expression of different GFP-tagged NSUN5 constructs. This suggests that either only small amounts of NSUN5 shuttle to the cytoplasm, or that extracts used for pulldowns might have contained small residual amounts of nuclear proteins and mRNAs. Anyhow, since RAS is an important stimulator of mTORC1 activity (58), it thereby promotes cytoplasmic translation and growth further downstream. Thus, subtle differences in RAS signalling and molecular function between different organisms might partially explain why loss of NSUN5 impairs growth in mice and human cells, but not in yeast, worm and fly (12).

We previously reported that NSUN5 orthologs modulate the chronological lifespan of yeast, as well as organismal lifespan of *C. elegans* and *D. melanogaster*. Importantly, this lifespan extension was conditional to reduced nutrient availability and was not accompanied by any obvious phenotypical alterations, such as body size, locomotion or feeding behaviour (12). *C. elegans* and *D. melanogaster* are predominantly post-mitotic organisms, thus impaired proliferation of cells is unlikely to affect organismal fitness and depletion of Rcm1 in yeast only extended chronological, but not replicative lifespan (12). Further experiments with our Nsun5 knockout mouse model will clarify, if loss of Nsun5 is able to modulate healthy lifespan of a complex model organism with characteristics of both chronological and replicative ageing.

In contrast to the small model organisms, we here discovered that loss of NSUN5 or catalytic mutation impairs ribosome function and reduces size and proliferation in mammalian cells, as well as the body weight of mice. These disagreements might be due to important differences between the models. NSUN5 depletion reduced bulk protein translation in mammalian cells, while a similar response was only evoked in yeast and *C. elegans* by exposure to stress such as hydrogen peroxide (12) or high salt concentrations (13,43). This might indicate that loss of m^5^C3782 renders ribosomes instable, which yeast and nematodes in contrast to mammalian cells are able to compensate under unstressed conditions.

Also, cells with high proliferation rates, such as cancer cells (HeLa, certain glioma cell lines under oxidative stress (15)) and immortalized cell lines (MEF), seem to be sensitive to loss of NSUN5, while in mice several normal tissues with low proliferation rates, including kidney, liver, lung and bone marrow were only mildly affected. Thus, we hypothesize that blunted expression of Nsun5, either by knockout or endogenous hypermethylation of its promoter, will mostly influence highly proliferative tissues of the adult mouse, which might explain the relatively mild general phenotype. Indeed, differential regulation of NSUN5 expression in various tissues was recently demonstrated to happen at epigenetic level, since methylation of the NSUN5 promoter was high in some glioma cell lines and primary tumors, but low in cervical and other malignancies (15). Thus, it will be interesting to determine epigenetic silencing of the NSUN5 locus also in various normal tissue contexts.

Importantly, the ancestral gene NSUN5, which we here investigated, has two additional copies (NSUN5B and NSUN5C) in the same chromosomal region related to WBS. All three gene products are described to be ubiquitously expressed and showing tissue-specific patterns (18). Weak expression of NSUN5 and high background staining of available antibodies did not allow us to assess which of the predicted genes are indeed expressed. However, only full-length NSUN5 is likely to participate in methylation of rRNA, because NSUN5B and NSUN5C roughly correspond to GFP-tagged truncation mutants, which did not localize to nucleoli. However, we cannot rule out specific functions of these genes in the cytoplasm or in another tissue context.

One of the main features of WBS is a mild to moderate cognitive impairment, including reduced cerebral volume by 13% (59,60). Recently, Zhang and coworkers described a novel Nsun5 knockout mouse model in the same C57BL/6J genetic background as our Nsun5 knockout mice. They demonstrated that loss of Nsun5 led to deficits in spatial learning and memory capacity, which might be connected to WBS pathology (20,21). Our data presented here further support this hypothesis in several ways: (i) Reduced proliferation of oligodendrocyte precursor cells in Nsun5 knockout mice (21) is most likely a general consequence of Nsun5 depletion, as we observed similar proliferation deficits in several other primary and immortalized cell types, (ii) alterations of ribosomes and protein translation, which we also observed upon NSUN5 loss, are frequently linked to other neuronal pathologies (61,62), and (iii) we were able to demonstrate that primary fibroblasts from WBS patients indeed showed decreased NSUN5 mRNA and protein levels resulting in a reduction of m^5^C3782, which clearly indicates that gene-dosage by haploinsufficiency of the NSUN5 gene in WBS is relevant. However, further studies with other cell types, more donors and advanced disease models, such as mice heterozygous for the Nsun5 gene, are required to draw definite conclusions and identify further symptoms that might be linked to NSUN5 haploinsufficiency. Better understanding of these molecular mechanisms will then potentially allow the development of novel therapeutics targeting the cognitive phenotype of WBS.

## Supplementary Material

gkz1043_Supplemental_File
