# Supplementary material for: Loss of the ribosomal RNA methyltransferase NSUN5 impairs global protein synthesis and normal growth
Source: Nucleic Acids Res. 2019 Nov 13;47(22):11807–25. doi: 10.1093/nar/gkz1043 (PMC7145617; doi:10.1093/nar/gkz1043)
Supplement: gkz1043_Supplemental_File [file gkz1043_supplemental_file.pdf]

Supplementary Material

Loss of the ribosomal RNA methyltransferase NSUN5 impairs global protein synthesis and normal growth

Clemens Heissenberger, Lisa Liendl, Fabian Nagelreiter, Yulia Gonskikh, Guohuan Yang, Elena M. Stelzer, Teresa L. Krammer, Lucia Micutkova, Stefan Vogt, David P. Kreil, Gerhard Sekot, Emilio Siena, Ina Poser, Eva Harreither, Angela Linder, Viktoria Ehret, Thomas H. Helbich, Regina Grillari-Voglauer, Pidder Jansen-Dürr, Martin Koš, Norbert Polacek, Johannes Grillari, Markus Schosserer

Contents:

Figure S1. Related to Figure 1 and Figure 5B: Full-length protein sequence alignment of Rcm1p and the human NSUN protein family 2

Figure S2. Related to Figure 2: CRISPR-Cas9 mediated gene editing of the NSUN5 locus reduced NSUN5 mRNA expression in HeLa 4

Figure S3. Related to Figure 2: Generation of stable HeLa and NSUN5 KO cell lines expressing endogenous levels of GFP-tagged mouse Nsun5 6

Figure S4. Related to Figure 2: Loss of NSUN5 decreases proliferation of primary cells and body weight of mice 8

Figure S5. Related to Figure 4: shRNA-mediated knockdown of NSUN5 in HEK293 cells indicates a trend towards impaired C3782 methylation 10

Figure S6. Related to Figure 5: RNA polymerase I activity targets NSUN5 to the nucleolus 12

Figure S7. Related to Figure 6: Quantification of mature ribosomes and analysis of ribosome biogenesis did not reveal changes upon loss of NSUN5 14

Supplementary experimental procedures 18

Supplementary references 25

Figure S1. Related to Figure 1 and Figure 5B: Full-length protein sequence alignment of Rcm1p and the human NSUN protein family

The full-length multiple protein sequence alignment of Rcm1p and the NSUN family (NSUN1 – NSUN7) is depicted. Colours indicate similarities. Two highly conserved cysteins required for covalent RNA substrate binding and subsequent release are indicated by asterisks (*). Protein domains of NSUN5 are highlighted by colours, as specified in the included legend.

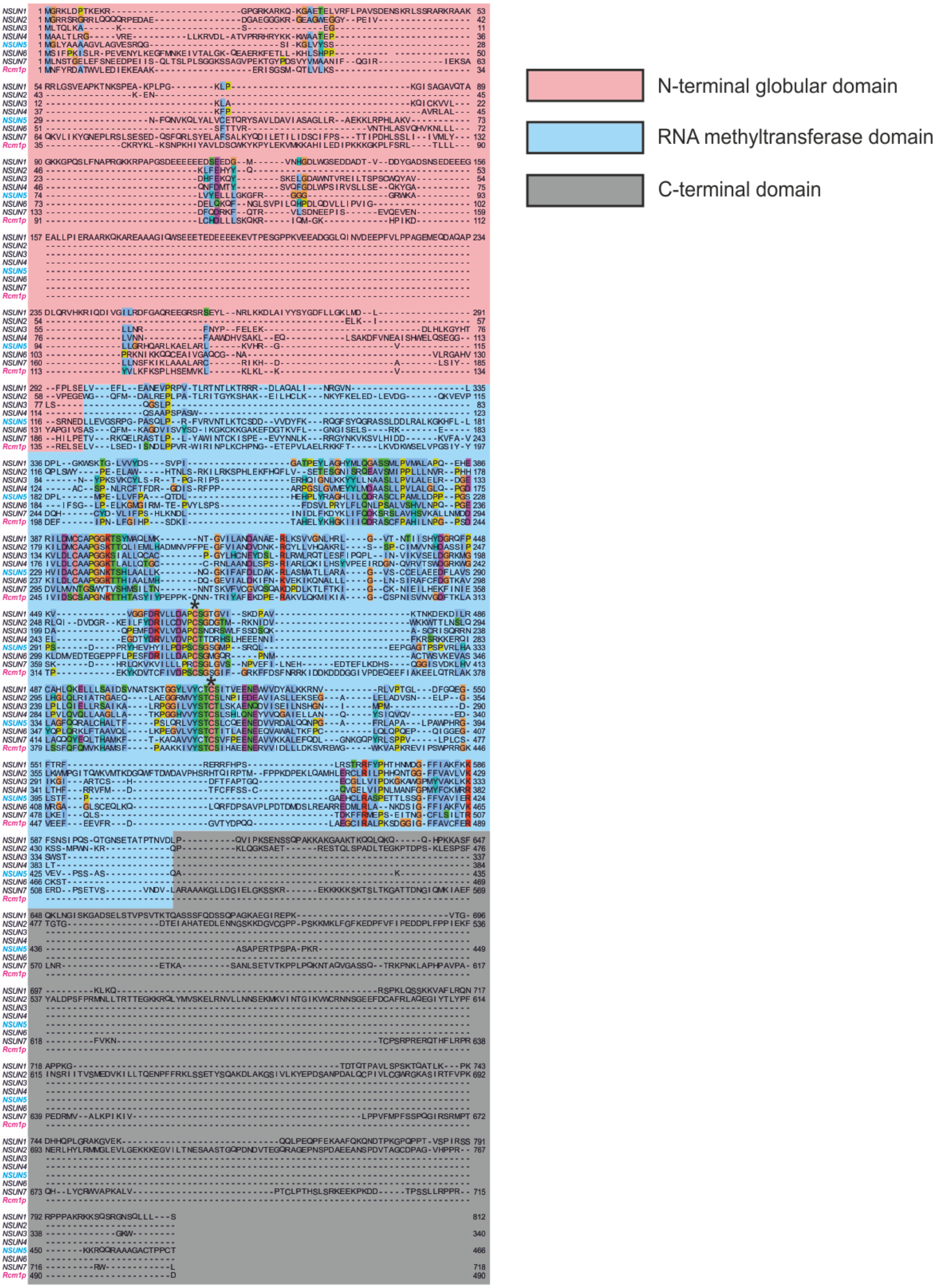
N-terminal globular domain
RNA methyltransferase domain
C-terminal domain

Figure S2. Related to Figure 2: CRISPR-Cas9 mediated gene editing of the NSUN5 locus reduced NSUN5 mRNA expression in HeLa

A, Sanger sequencing of genomic DNA from NSUN5 KO cells reveals translocation from genomic region 4q into the guide-RNA (gRNA) target site between intron 1 and exon 2 of the NSUN5 locus. An alignment of nine TOPO-sequencing clones with the NSUN5 reference sequence (*NSUN5_reference.seq*) is shown. Introns are highlighted in green, exons in orange and the gRNA in grey. **B**, NSUN5 mRNA expression was measured by RT-qPCR of HeLa and NSUN5 KO cells and showed decreased NSUN5 mRNA expression in NSUN5 KO cells. Error bars indicate standard deviation. n = 3 independent replicates. *$p < 0.05$, Student´s t-test.

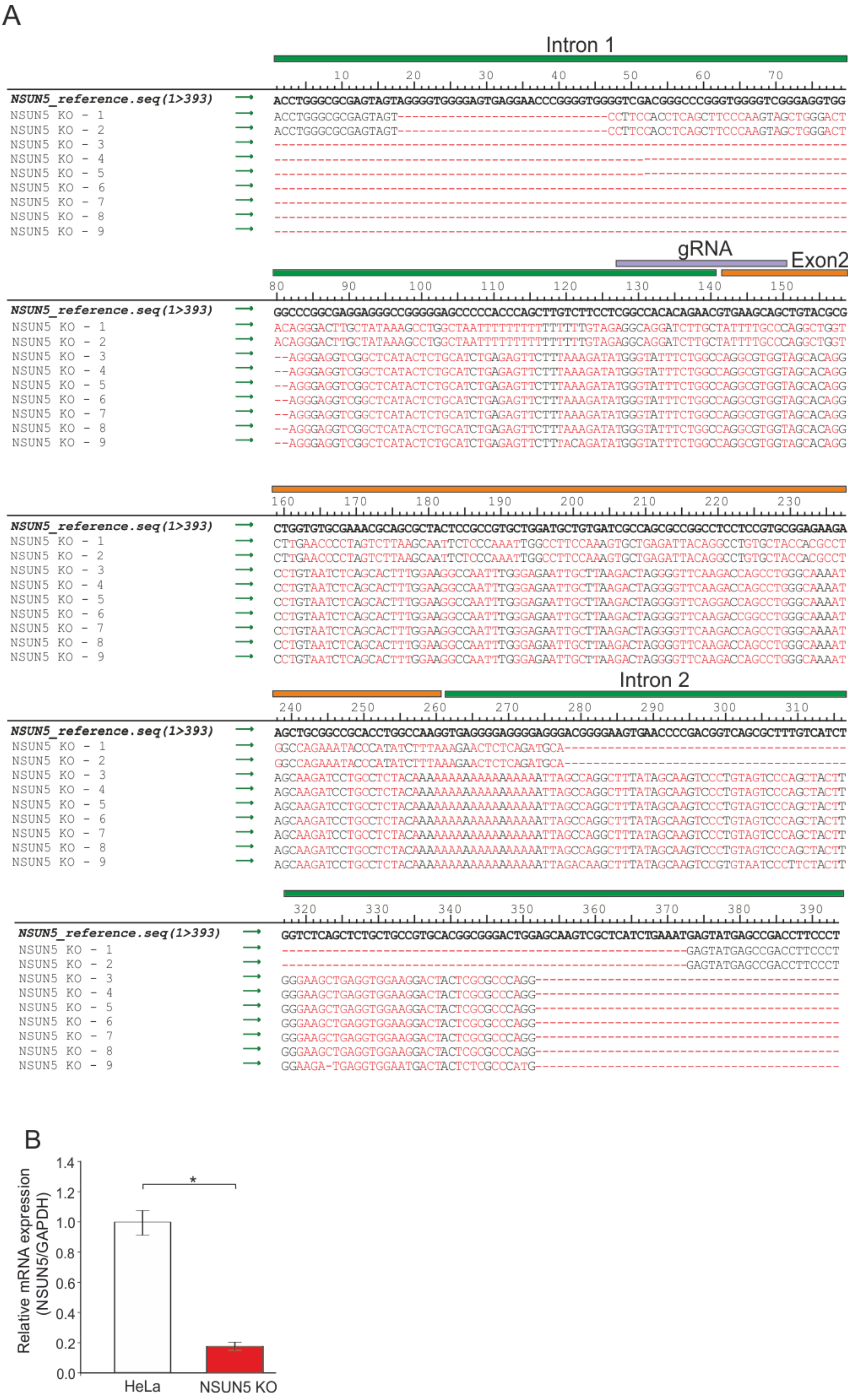
A
Intron 1
gRNA
Exon2
Intron 2
NSUN5_reference.seq(1>393)
NSUN5 KO - 1
NSUN5 KO - 2
NSUN5 KO - 3
NSUN5 KO - 4
NSUN5 KO - 5
NSUN5 KO - 6
NSUN5 KO - 7
NSUN5 KO - 8
NSUN5 KO - 9
B
Relative mRNA expression (NSUN5/GAPDH)
1.4
1.2
1.0
0.8
0.6
0.4
0.2
0.0
*
HeLa
NSUN5 KO

Figure S3. Related to Figure 2: Generation of stable HeLa and NSUN5 KO cell lines expressing endogenous levels of GFP-tagged mouse Nsun5

A, Western blot of GFP-Nsun5 constructs shows screening and selection of stable cell lines expressing BACmid-encoded GFP-mNsun5 in HeLa and NSUN5 KO cells. HeLa transiently expressing GFP (HeLa + pEGFP-N1) were included as control. Asterisks (*) indicate selected cell lines. Probing with α-GFP confirms the expected size of GFP-mNsun5 at around 80 kDa and of free GFP at 25 kDa. **B**, Fluorescence microscopy of HeLa GFP-mNsun5 and NSUN5 KO GFP-mNsun5 indicates localization of GFP-mNsun5 in nucleoli and nucleoplasm. Arrows indicate nucleoli. Scale bars represent 15 µm.

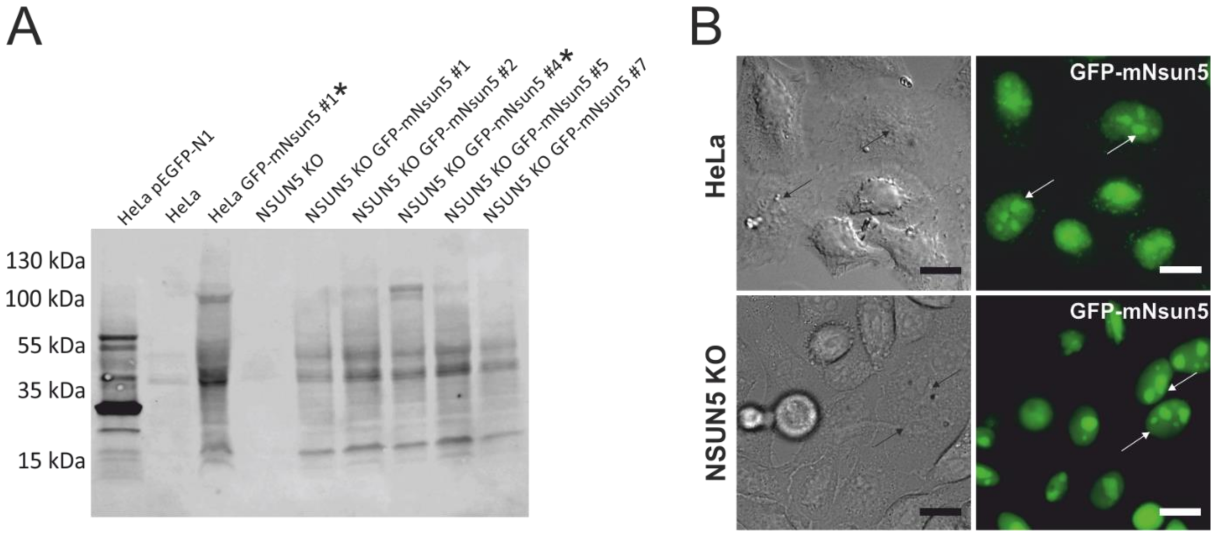
A
HeLa pEGFP-N1
HeLa
HeLa GFP-mNsun5 #1*
NSUN5 KO
NSUN5 KO GFP-mNsun5 #1
NSUN5 KO GFP-mNsun5 #2
NSUN5 KO GFP-mNsun5 #4*
NSUN5 KO GFP-mNsun5 #5
NSUN5 KO GFP-mNsun5 #7
130 kDa
100 kDa
55 kDa
35 kDa
15 kDa
B
HeLa
NSUN5 KO
GFP-mNsun5
GFP-mNsun5

Figure S4. Related to Figure 2: Loss of NSUN5 decreases proliferation of primary cells and body weight of mice

A, RT-qPCR reveals knockdown of NSUN5 mRNA in fibroblasts upon transduction with four different shRNAs against NSUN5, compared to a non-hairpin forming control (SCR). **B**, Cell numbers of shRNA-transduced fibroblast strains were recorded after selection of stable transformants (10 – 31 days post transduction) and normalized to the SCR control. n = 3 independent experiments with strains from three different donors. *$p \leq 0.05$, n.s. = not significant, one sample t-test against expected value of 1. **C**, SCR, sh#3 and #4 of one replicate (squares in panel B) were re-seeded at equal cell numbers after selection of stable transformants (10 days post transduction) and counted 10 days after re-seeding. **D**, The design of the Nsun5 knockout mouse model is depicted. Exon 1, intron 1 and parts of exon 2 were replaced by two LoxP sites. **E**, Western blot confirms loss of Nsun5 protein expression in kidneys of wildtype (+/+) and knockout (-/-) mice. GAPDH was used as loading control. The blot was probed with Santa Cruz α -NSUN5. **F**, RT-qPCR confirms loss of Nsun5 mRNA expression. Data were normalized to Actb. Error bars indicate standard deviation. $n \geq 3$ animals. ***$p < 0.005$. One-Way Anova followed by post -hoc Dunnet´s test.

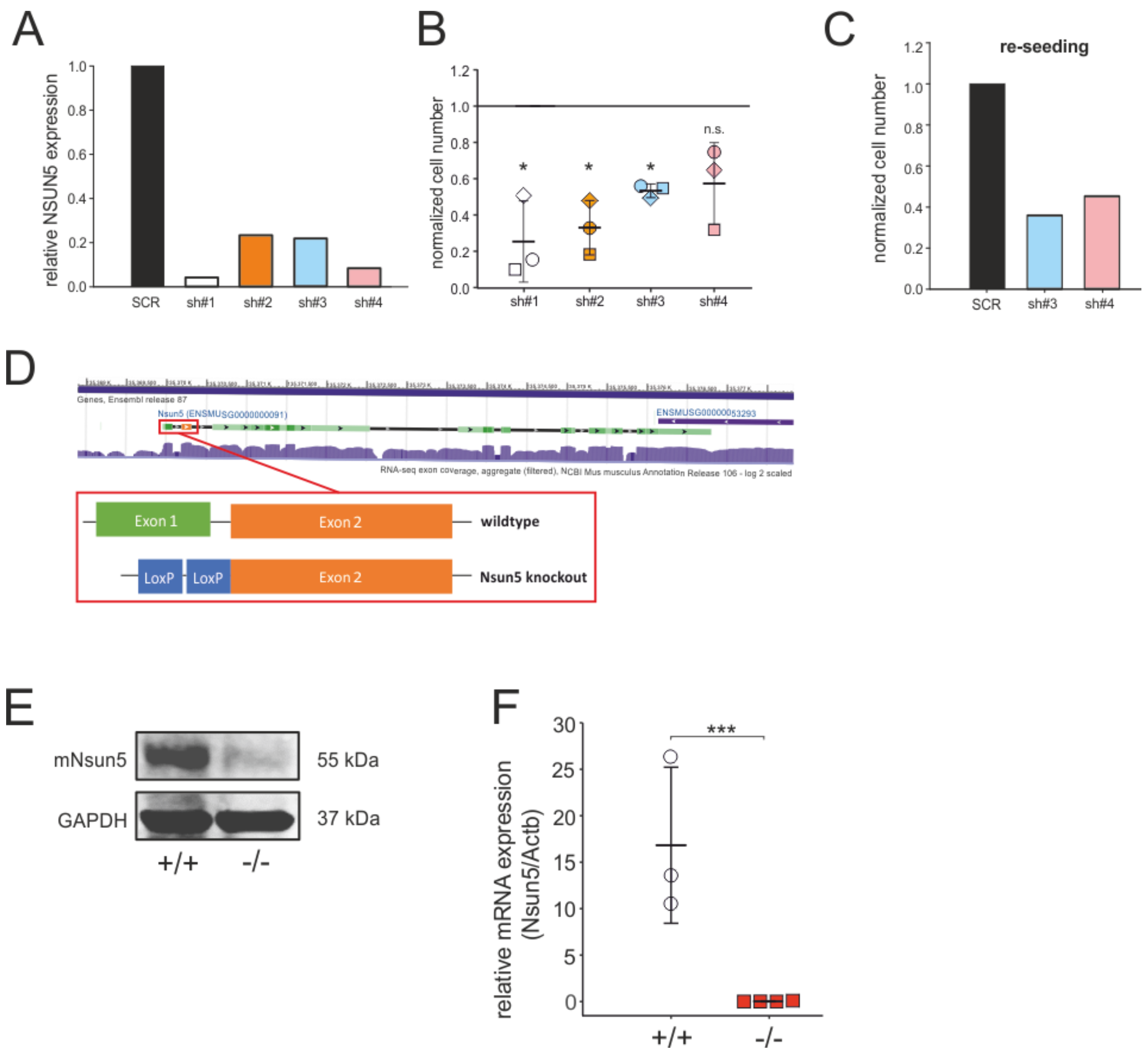
A
relative NSUN5 expression
1.0
0.8
0.6
0.4
0.2
0.0
SCR
sh#1
sh#2
sh#3
sh#4
B
normalized cell number
1.2
1.0
0.8
0.6
0.4
0.2
0.0
*
*
*
n.s.
sh#1
sh#2
sh#3
sh#4
C
re-seeding
normalized cell number
1.2
1.0
0.8
0.6
0.4
0.2
0.0
SCR
sh#3
sh#4
D
Genes, Ensembl release 87
RNA-seq exon coverage, aggregate (filtered), NCBI Mus musculus Annotation Release 106 - log 2 scaled
Exon 1
Exon 2
wildtype
LoxP
LoxP
Exon 2
Nsun5 knockout
E
mNsun5
55 kDa
GAPDH
37 kDa
+/+
-/-
F
relative mRNA expression
(Nsun5/Actb)
30
25
20
15
10
5
0
***
+/+
-/-

Figure S5. Related to Figure 4: shRNA-mediated knockdown of NSUN5 in HEK293 cells indicates a trend towards impaired C3782 methylation

A, COBRA assay indicates a trend towards decreased C3782 methylation in HEK293 cells upon shRNA-mediated knockdown of NSUN5. Arrow depicts the 45 bp fragment, representing methylation and the asterisk (*) indicates the 29 bp fragment, representing non-methylation. **B**, Quantification of the COBRA assay. n = 3 independent experiments. One-way Anova followed by Dunnet´s post-hoc test does not reveal statistical significance. **C**, Relative expression levels of NSUN5 mRNA were determined by RT-qPCR. n = 3 independent experiments. One-way Anova followed by Dunnet´s post-hoc test does not reveal statistical significance.

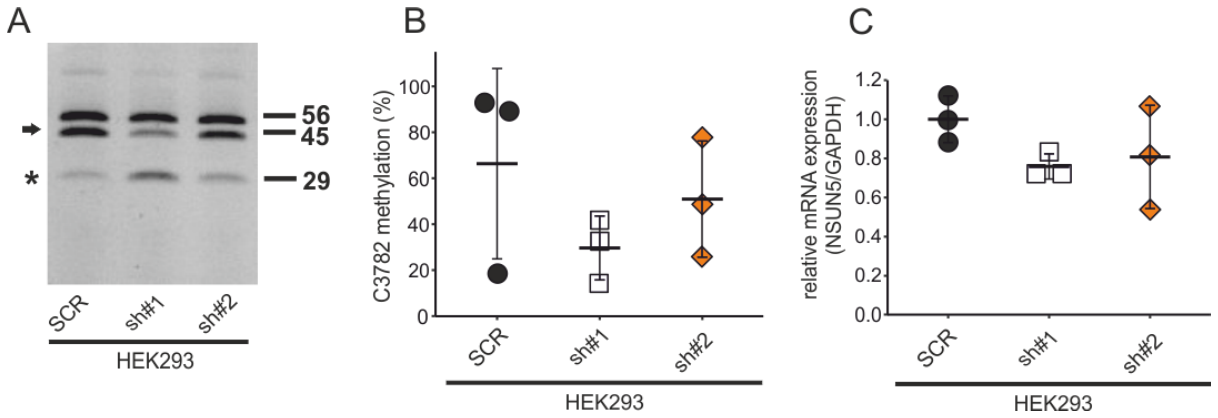
A
56
45
29
*
SCR
sh#1
sh#2
HEK293
B
C3782 methylation (%)
100
80
60
40
20
0
SCR
sh#1
sh#2
HEK293
C
relative mRNA expression
(NSUN5/GAPDH)
1.2
1.0
0.8
0.6
0.4
0.2
0.0
SCR
sh#1
sh#2
HEK293

Figure S6. Related to Figure 5: RNA polymerase I activity targets NSUN5 to the nucleolus

HeLa cells were exposed to 50 µg/µL α-Amanitin to block RNA Polymerase II and III or 50 ng/µL Actinomycin D to block RNA Polymerase I, II and III. NSUN5 (green) and Fibrillarin (red) were visualized by indirect immunofluorescence staining. Fluorescence microscopy reveals nucleolar localization of NSUN5 and segregation to nucleolar caps upon inhibition of RNA polymerase I. Nuclei were counterstained with DAPI (blue). Images were processed by deconvolution and brightness and contrasts were adjusted. Scale bar represents 15 µm.

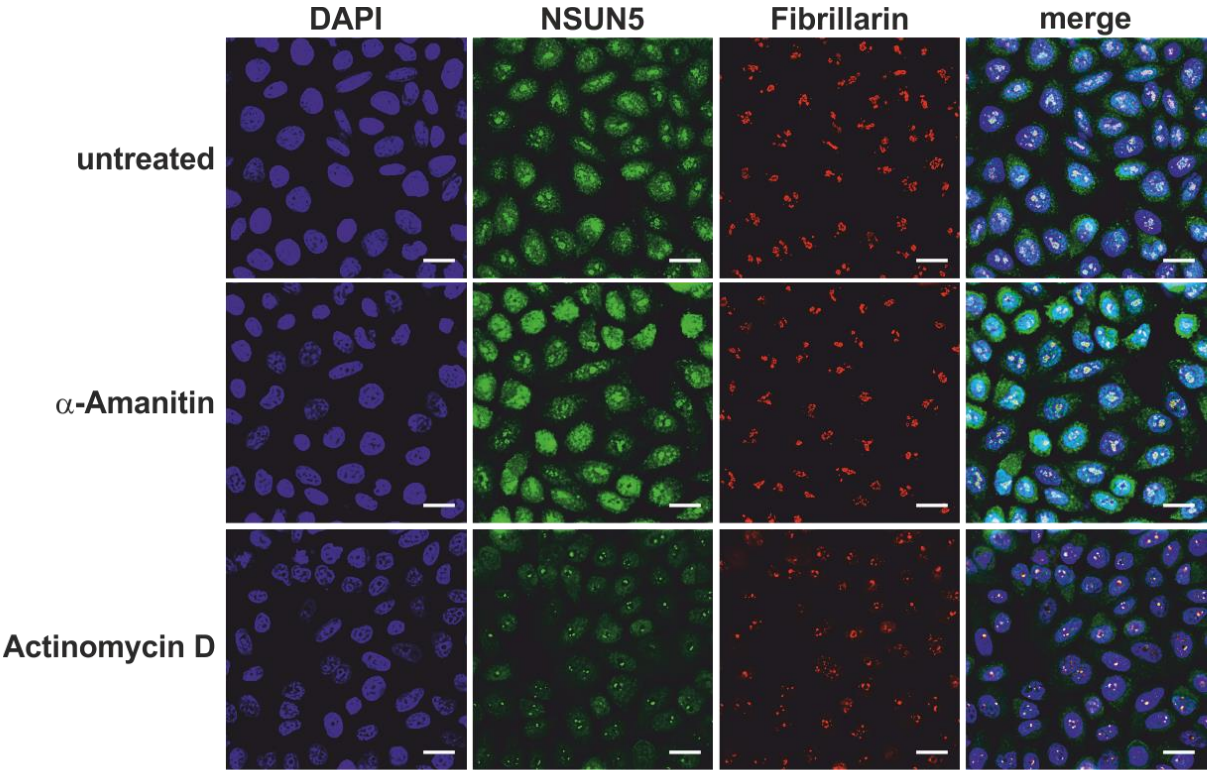
DAPI
NSUN5
Fibrillarin
merge
untreated
α-Amanitin
Actinomycin D

Figure S7. Related to Figure 6: Quantification of mature ribosomes and analysis of ribosome biogenesis did not reveal changes upon loss of NSUN5

A, Quantification of 28S and 18S rRNA fragments show no differences in mature ribosomes of HeLa compared to NSUN5 KO cells. n = 2 independent experiments with 2 technical replicates each. **B,** Scheme of human ribosome biogenesis. **C-E,** Northern blots using two specific pre-rRNA probes, ITS1 (**C**) and ITS2 (**D**), indicate no alterations in ribosome biogenesis in HeLa compared to NSUN5 KO cells. Methylene blue staining of 28S and 18S rRNA confirms equal loading (**E**). n = 3 replicates. **F-I,** Dual luciferase reporter assays show no statistically significant difference in translational fidelity upon loss of NSUN5 in HeLa cells as measured by read-through of all three stop codons (**F**), -1 frameshifting induced by viral sequences (FS -1 (L-A) and FS -1 (HIV)) (**G**), as well as amino acid misincorporation into the first (K529E), second (K529I) and third (K529N) codon position (**H**). Normalized luminescence relative to the respective control plasmids is shown. n = 3 independent replicates. Error bars indicate standard deviation. Two sample t-tests of control HeLa vs. NSUN5 KO. **I**, IRES-dependent translation as measured by a dual luciferase reporter assay does not reveal a significant difference between control HeLa and NSUN5 KO cells. n = 3 independent replicates. Error bars indicate standard deviation. Two sample t-tests of control HeLa vs. NSUN5 KO.

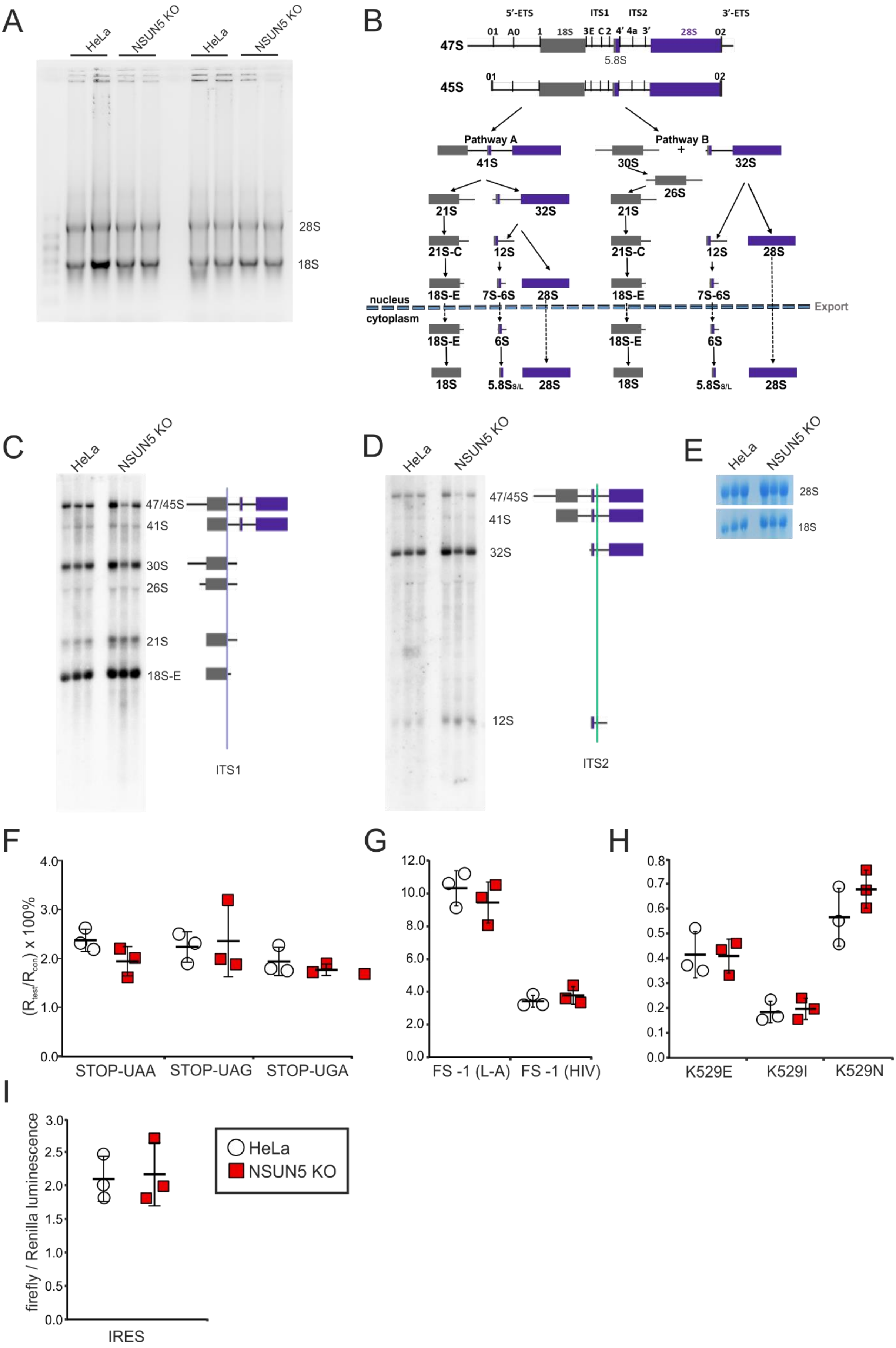
A
HeLa
NSUN5 KO
28S
18S
B
5'-ETS
ITS1
ITS2
3'-ETS
47S
45S
5.8S
Pathway A
Pathway B
41S
30S
26S
32S
21S
21S-C
12S
28S
18S-E
7S-6S
nucleus
cytoplasm
Export
6S
18S
5.8SS/L
C
47/45S
41S
30S
26S
21S
18S-E
ITS1
D
32S
12S
ITS2
E
F
(R_{test}/R_{con}) x 100%
STOP-UAA
STOP-UAG
STOP-UGA
G
FS -1 (L-A)
FS -1 (HIV)
H
K529E
K529I
K529N
I
firefly / Renilla luminescence
IRES

Figure S8. Related to experimental procedures: Characterization of selfmade anti-NSUN5 antibody

A, Brightfield and fluorescence microscopy showed similar nucleolar staining of endogenous NSUN5 in HeLa cells with both commercial and selfmade NSUN5 antibodies. Arrows indicate nucleoli. Scalebars represent 15 µm. **B**, Western blot of a HeLa cell lysate probed with the selfmade α -NSUN5 antibody revealed a prominent band at ~60 kDa.

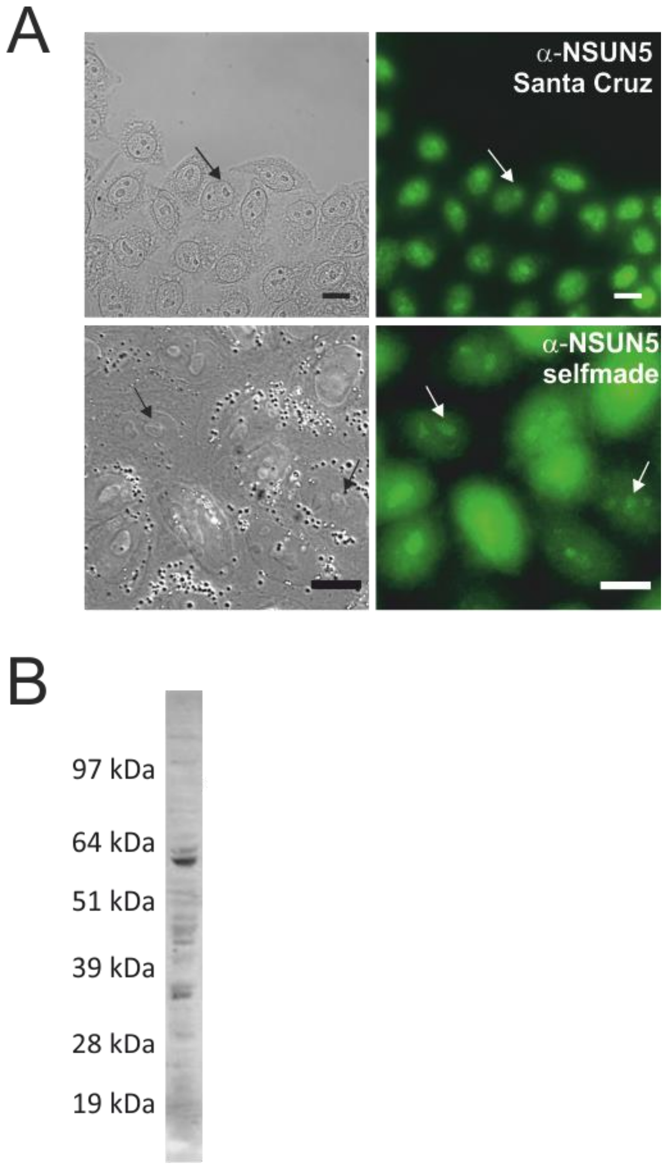
A
α-NSUN5
Santa Cruz
α-NSUN5
selfmade
B
97 kDa
64 kDa
51 kDa
39 kDa
28 kDa
19 kDa

Supplementary experimental procedures

Isolation and immortalization of mouse embryonic fibroblasts

Embryos from homozygous mating were isolated between E 12.5 and E 14.5. After removing the heads, embryos were minced and incubated with trypsin-EDTA (0.25%) at 37 °C for 30 minutes. After filtering the suspension through a cell strainer (100 µm) and harvesting cells via centrifugation, cells were seeded in cell culture flasks. For immortalization, cells were passaged every three days and 12.000 cells per cm^2 were seeded. After about 20 passages when cells passed crisis stage, they were passaged at a 1:10 split ratio. Mouse embryonic fibroblasts (MEFs) were cultured in DMEM (F0435, Biochrom), supplemented with 10% FCS, 4 mM L-glutamine and 0.1 mM β-mercaptoethanol.

Transfection of mammalian cells

Cells were grown to ~80% confluence in 6-well cell culture plates. 2 µg of plasmid DNA were mixed with 4 µL of jetPRIME transfection reagent (Polyplus transfection), vortexed and incubated for 10 minutes. The mixture was added dropwise to the cells. Culture medium was exchanged 16 – 24 h after transfection. Lentiviruses containing NSUN5, C308S and C359S were produced by co-transfecting Lenti-X HEK-293T cells with the recombinant pLVX-IRES-Hyg plasmid and a Lenti-X Packaging Single Shot (Takara) following the supplier instructions. The transfection cocktail was removed after 24 h and replaced with fresh medium. 72 h after transfection, supernatant containing viral particles was harvested 0.45 µm filtered and stored at -80 °C before transduction. Presence of virus particles was tested using Lenti-X GoStix Plus (Takara). NSUN5 KO HeLa -and MEF -/- cells were infected with virus particles. One day after infection, antibiotic selection (400 µg/mL hygromycin) was initiated. After 5 days of selection, stably expressing cells were expanded and passaged at least three times before starting experiments in media containing 200 µg/mL hygromycin.

NSUN5 knock-down by shRNA

The following shRNA constructs in the pLKO.1 vector, as well as a non-hairpin forming construct as negative control (1), were acquired from Open Biosystems: Non hairpin control (CCGCAGGTATGCACGCGT), NSUN5 shRNA #1 (AGGCAATAAGACCAGTCACT), NSUN5 shRNA #2 (AAGAGACAAGGTTTCTCCTA), NSUN5 shRNA #3 (CAAGGGAAGATCTTTGCCTT) and NSUN5 shRNA #4 (CAAGGTGCTAGTGTATGAGT).

Lentiviral particles were packaged by transfecting 293FT cells as previously described (2). 293FT cells were seeded into T75 cell culture flasks at cell numbers that resulted in 90-95% confluence on the day of transfection. For transfection, the culture medium was removed and cells were washed once with 10 mL PBS. 5 mL of growth medium without antibiotics were added. In a sterile 15 mL tube, 7.5 µg psPAX packaging plasmid, 2.5 µg pMD2.G envelope plasmid and 3 µg of shRNA pLKO plasmid DNA were diluted in 1.5 mL Opti-MEM I Medium (Life Technologies). In a separate sterile tube, 36 µL Lipofectamine 2000 (Life Technologies) were diluted in 1.5 mL of Opti-MEM I Medium. The Lipofectamine/Opti-MEM I suspension was gently mixed and incubated for 5 minutes at room temperature. After 5 minutes incubation, the diluted DNA was combined with diluted Lipofectamine 2000. The solution was gently mixed and incubated for 20 minutes at room temperature to allow the DNA / Lipofectamine 2000 complexes to form. The DNA / Lipofectamine 2000 complexes were added to the cells and incubated overnight. The next day, medium was exchanged. Viruses were harvested after 48 h by centrifugation of the supernatant at 300 g for 10 minutes to remove debris. The viral supernatant was filtered through a Millex-HV 0.45 µm filter (Merck-Millipore), aliquoted into cryo tubes and stored at -80 °C. An extra vial with 10 µL of virus was frozen for quantitation of the infection titer.

For the transduction of HEK293 and HDF, 50,000 cells were seeded into one well of a 6-well plate and incubated overnight. A multiplicity of infection (MOI) of four was used with the addition of 8 µg/mL Polybrene to the medium. The next day, medium was exchanged. 3 days after transduction puromycin (500 ng/mL) was added to the medium and the medium containing puromycin was exchanged every

three days. After 6-8 days the selection was finished and the knockdown efficiency was evaluated by RT-qPCR and western blot.

Western blots

Cells were lysed in RIPA-buffer (150 mM NaCl, 1% NP-40, 0.5% sodium deoxycholate, 0.1% SDS, 50 mM Tris/HCl pH 8.0), sonicated for 30 cycles (30 s on / 30 s off) with a Bioruptor Plus sonicator (Diagenode) and mixed with SDS-PAGE sample buffer (60 µM Tris/HCl pH 6.8, 2% SDS, 10% glycerol, 0.0125% bromophenol blue and 1.25% β-mercaptoethanol). Mouse kidneys were cut in small pieces, lysed in RIPA-buffer, sonicated for 30 cycles (30 s on / 30 s off) and centrifuged briefly. The supernatant was mixed with SDS-PAGE sample buffer.

Lysates were heated to 95 °C for 10 min and cooled on ice. Electrophoresis was performed using 4-15% Mini-PROTEAN® TGX Gels (BioRad) in Laemmli-Buffer (25 mM Tris, 250 mM glycine and 0.1% SDS) (3) at 150 V for approximately one hour. The protein bands were transferred from SDS-PAGE gels to a PVDF-membrane (Bio Rad) at 25 V and 1.3 A for 3 min. Afterwards the membrane was incubated for 1 h in Blocking Buffer (PBS containing 0.1% Tween-20 and 3% non-fat dry milk), followed by primary antibodies in Blocking Buffer for either one hour at room temperature or overnight at 4 °C. After three washes with PBS containing 0.1% Tween-20 for 5 min each, secondary antibodies in Blocking Buffer were added and incubated for 1 h at room temperature. After three consecutive washes in PBS containing 0.1% Tween-20, and one final wash in PBS without Tween-20, detection was performed on the Odyssey Infrared Imager (LI-COR) at 700 and 800 nm.

Immunofluorescence staining of cells

Cells were seeded onto coverslips or into µ-slides (ibidi GmbH) and incubated over night at 37 °C. Afterwards the cells were fixed in 4% formaldehyde in PBS, washed two times with PBS for 5 min each, and permeabilized for 10 min in 1% Triton X-100 in PBS, followed by two PBS washing steps.

Primary and secondary antibodies were diluted in PBS containing 2% BSA. Samples were incubated with primary and secondary antibody solutions for 30 min respectively in a humidified chamber at room temperature, each followed by four washes in PBS. Nuclei were counterstained with DAPI and images were recorded on a Leica DMI6000B epifluorescence microscope using the A4, L5 and N2.1 filter cubes. Deconvolution with Huygens Essential version 4.0.0p5 64 b (Scientific Volume Imaging) was performed when indicated in the figure legend. Cropping, insertion of scale bars and brightness and contrast adjustments were done with Image J (Version 1.52 e).

Immunofluorescence staining for gSTED microscopy was performed as described above with the following modifications: After incubation with primary antibodies and the washing, cells on coverslips were stained with Oregon Green 488 anti-mouse and Biotin anti-rabbit antibodies. After the final wash, coverslips were incubated with V500-streptavidin (Becton Dickinson) 1:250 in 2% BSA in PBS for 30 min, washed three times with PBS and mounted with Mowiol (Sigma) on slides. Counterstaining with DAPI was omitted. On the next day when Mowiol was hardened, imaging was performed on a SP8 confocal microscope equipped with a pulsed white-light laser and gSTED (Leica Microsystems) with the following parameters: objective: HC PL APO CS2 100x/1.40 OIL, bi-directional scan at 400 Hz, pinhole = 1 Airy unit, frame accumulation = 6, line average = 6, sequential accumulation: 503 nm pulsed laser excitation / time-gated hybrid detector at 533 nm – 575 nm / 592 nm STED depletion for NSUN5 and 470 nm pulsed laser excitation / time-gated hybrid detector at 479 nm – 511 nm / 592 nm STED depletion for Fibrillarin. Deconvolution of images was performed with Huygens Professional (Scientific Volume Imaging).

O-propargyl-puromycin (OPP) assay

OPP assays were performed as described previously (4). In brief, cells were grown to ~80% confluence and incubated with 25 µM OPP (#NU-931-05, Jena Bioscience) for 20 minutes at 37 °C, 7% CO_2. After harvesting, cells were fixed by adding 5 mL ice-cold 70% ethanol with gentle agitation on a vortex-mixer to avoid clumping. Two specificity controls were included: 1) cells not labelled with OPP and 2)

cells incubated with cycloheximide (#C7698, Sigma) at a final concentration of 50 µg/mL for 15 min prior to, as well as during incubation with OPP.

In order to visualize OPP incorporation by a fluorescent label, cells were pelleted and resuspended in 1 mL "Click Chemistry Buffer" (115 mM Tris/HCl pH 8.5, 0.1% Triton X-100). After another centrifugation, cells were re-suspended in 500 µL freshly prepared "Click Chemistry Mix" (500 µL "Click Chemistry Buffer", 10.6 µL 100 mM $CuSO_4$ solution, 53.3 µL 20 mg/mL ascorbic acid solution and 1 µL 10 mM Alexa Fluor 647 azide) and incubated for 30 min at room temperature protected from light. Subsequently, cells were washed twice with 1 mL "Wash Buffer" (100 mM Tris/HCl pH 7.4, 2 mM $MgCl_2$, 0.1% Triton X-100). All centrifugation steps were performed at 400 g for 5 min.

For quantitation of OPP labelling, cells were analysed by flow cytometry (Gallios flow cytometer; Beckman Coulter), using a 638 nm red diode 25 mW laser for the analysis of AF647 signals (FL6, 660/10 bandpass filter). Selection of whole single cells in G1 phase was based on forward and side scatter, as well as on DAPI staining. Analysis of fluorescence intensities was performed in Kaluza 1.2 (Beckman Coulter).

Quantification of rRNA

28S and 18S rRNA were separated on a 1% agarose gel from 400 µg input RNA. 28S and 18S rRNA were then electroeluted into a dialysis bag (Spectra/Por #132720). After dialysis, RNA samples were precipitated and again loaded on a gel. Fragments were treated as described above and finally eluted with distilled water. 5.8S and 5S rRNA of 400 µg input RNA were separated on an 8% polyacrylamide gel containing 7 M urea. 5.8S and 5S rRNA were extracted, eluted and reloaded on a gel as described above. Fragments were finally eluted with distilled water.

Analysis of ribosome biogenesis by northern blot

4 µg purified total RNA were separated on a 1.2% agarose gel. Separated RNA was then transferred to a wet nylon membrane using electro-transfer at 4 °C. Crosslinking was performed in a UV-Crosslinking device (Hoefer UVC 500 Ultraviolet Crosslinker) utilizing a 254 nm light source for 30 seconds. Afterwards, the membrane was stained with methylene blue to visualize 18S and 28S rRNA. Probes (ITS1: 5´ GTCCGGGCTCCGTTAATGATC ´3 and ITS2: 5´ GGCAAGAGGAGGGCGGA ´3) were hybridized with the membrane at 38 °C to 42 °C overnight. Then membranes were exposed to imaging plates (Fuji). Signal intensities were quantified, and background was subtracted (Fuji FLA-5100 scanner and AIDA software, Raytest).

Reporter assays to characterize translational fidelity and IRES translation

pGL3 (WT), pGL3 (E - K529E), pGL3 (I - K529I) and pGL3 (N - K529N) to test amino-acid misincorporation were a kind gift from Vera Gorbunova. The pcDNA3 (RLUC POLYIRES FLUC) plasmid to test IRES-dependent translation was a kind gift from Nahum Sonenberg (5). pCI-neo (control), pCI-neo (FS -1 (L-A)), pCI-neo (FS -1 (HIV)), pCI-neo (Stop-UAA), pCI-neo (Stop-UAG) and pCI-neo (Stop-UGA) to measure stop-codon read through and translational frameshifting were generated as follows:

The dual luciferase reporter constructs to analyse translational frameshifting were generously provided by Jonathan Dinman (6), transferred into CMV-promoter containing yeast expression vectors to generate pMK967 (control), pMK968 (FS -1 (L-A)), pMK970 (FS -1 (HIV)), pMK971 (Stop-UAA), pMK972 (Stop-UAG) and pMK973 (Stop-UGA), and further cloned into the mammalian expression vector pCI-neo. For this aim, the DNA sequence of the luciferase fusion constructs were amplified by polymerase chain reaction from pMK plasmids using Q5-Polymerase (New England Biolabs) and the CLF forward primer introducing a recombination site and the KOZAK initiation sequence at the 5′ end (5′-CTAGCCTCGAGAATTCGCCGCCACCATGACTTCGAAAGTTTATGATCCAGAACAAAG-3′), as well as the CLR reverse primer appending another recombination site at the 3′ end (5′-

TACCACGCGTGAATTCTTACAATTTGGACTTTCCGCCCTTCTTG-3′). The pCI-neo vector backbone was linearized by restriction digest with EcoRI-HF (New England Biolabs). Purified inserts and linearized vector DNA were consequently recombined using the In-Fusion cloning system (Takara Bio USA Inc.) according to the manufacturer's instructions.

The firefly luciferase plasmids pGL3 (WT), pGL3 (E - K529E), pGL3 (I - K529I), pGL3 (N - K529N) and pcDNA3 (RLUC POLYIRES FLUC) were mixed with pGL3 coding for Renilla luciferase for normalization in a 4:1 ratio and transfected into mammalian cells as described above. The dual-luciferase plasmids pCI-neo (control), pCI-neo (FS -1 (L-A)), pCI-neo (FS -1 (HIV)), pCI-neo (Stop-UAA), pCI-neo (Stop-UAG) and pCI-neo (Stop-UGA) were transfected without mixing with another plasmid. 48 h after transfection, translational fidelity of transfected cells was analysed by using the Dual-Luciferase assay kit (Promega). Cells were harvested by scraping in lysis buffer (25 mM Tris-HCl pH 7.8, 1% Triton X-100, 0.1% SDS, and 0.5% sodium deoxycholate) and incubation for 10 minutes at room temperature. Extracts were thoroughly mixed with luciferase reagent and luminescence was measured after 20 min incubation at room temperature on a microplate reader (Tecan, Life Sciences). Samples were then mixed with Dual-Glo® Stop & Glo® reagent (blocking firefly luciferase and initiating Renilla luciferase), incubated for 20 min at room temperature and luminescence was measured. Relative luminescence [%] to quantify translational fidelity was calculated as previously described (6):

$$relative\ luminescence\ [\%] = \frac{\frac{firefly\ [sample]}{Renilla\ [sample]}}{\frac{firefly\ [control]}{Renilla\ [control]}} * 100$$

Supplementary references

1. Moffat,J., Grueneberg,D.A., Yang,X., Kim,S.Y., Kloepfer,A.M., Hinkle,G., Piqani,B., Eisenhaure,T.M., Luo,B., Grenier,J.K., *et al.* (2006) A lentiviral RNAi library for human and mouse genes applied to an arrayed viral high-content screen. *Cell*, **124**, 1283–1298.

2. Micutkova,L., Diener,T., Li,C., Rogowska-Wrzesinska,A., Mueck,C., Huetter,E., Weinberger,B., Grubeck-Loebenstein,B., Roepstorff,P., Zeng,R., *et al.* (2011) Insulin-like growth factor binding protein-6 delays replicative senescence of human fibroblasts. *Mech. Ageing Dev.*, **132**, 468–79.

3. Laemmli,U.K. (1970) Cleavage of structural proteins during the assembly of the head of bacteriophage T4. *Nature*, **227**, 680–685.

4. Nagelreiter,F., Coats,M.T., Klanert,G., Gludovacz,E., Borth,N., Grillari,J. and Schosserer,M. (2018) OPP Labeling Enables Total Protein Synthesis Quantification in CHO Production Cell Lines at the Single-Cell Level. *Biotechnol. J.*, **13**, e1700492.

5. Poulin,F., Gingras,A.C., Olsen,H., Chevalier,S. and Sonenberg,N. (1998) 4E-BP3, a new member of the eukaryotic initiation factor 4E-binding protein family. *J. Biol. Chem.*, **273**, 14002–7.

6. Harger,J.W. and Dinman,J.D. (2003) An in vivo dual-luciferase assay system for studying translational recoding in the yeast Saccharomyces cerevisiae. *RNA*, **9**, 1019–24.
